# Making Your Own Luck: Weak Vertical Swimming Improves Dispersal Success for Coastal Marine Larvae

**DOI:** 10.1007/s11538-023-01252-2

**Published:** 2024-01-28

**Authors:** Alexander D. Meyer, Alan Hastings, John L. Largier

**Affiliations:** 1 Department of Biological Sciences, University of Notre Dame, Notre Dame, IN USA; 2 Department of Mathematics, University of California, Davis, CA USA; 3 Department of Environmental Science and Policy, University of California, Davis, CA USA; 4 Santa Fe Institute, 1399 Hyde Park Road, Santa Fe, NM USA; 5 Coastal and Marine Sciences Institute, University of California Davis, 2099 Westshore Rd., Bodega Bay, CA USA

**Keywords:** Optimal control, Dynamic programming, Dispersal ecology, Marine ecology, Behavioral ecology

## Abstract

Dispersive early life stages are common in nature. Although many dispersing organisms (“propagules”) are passively moved by outside forces, some improve their chances of successful dispersal through weak movements that exploit the structure of the environment to great effect. The larvae of many coastal marine invertebrates, for instance, swim vertically through the water column to exploit depth-varying currents, food abundance, and predation risk. Several swimming behaviors and their effects on dispersal between habitats are characterized in the literature, yet it remains unclear when and why these behaviors are advantageous. We addressed this gap using a mathematical model of larval dispersal that scored how well behaviors allowed larvae to simultaneously locate habitats, avoid predators, and gather energy. We computed optimal larval behaviors through dynamic programming, and compared those optima against passive floating and three well documented behaviors from the literature. Optimal behaviors often (but not always) resembled the documented ones. However, our model predicted that the behaviors from the literature performed robustly well, if not optimally, across many conditions. Our results shed light on why some larval behaviors are widespread geographically and across species, and underscore the importance of carefully considering the weak movements of otherwise passive propagules when studying dispersal.

**Supplementary Information:**

The online version contains supplementary material available at 10.1007/s11538-023-01252-2.

## Introduction

Many plants, animals, and fungi have dispersive early life stages where individuals are transported relatively long distances by outside forces (Cowen and Sponaugle 2008; Levin et al 2003; Okubo and Levin 2001). Familiar examples include the wind-borne seeds of the common dandelion, *Taraxacum officinale* (Nathan et al 2008); the the hatchlings of ballooning spiders that sail through the air on gossamer parachutes (Suter 1999); and the planktonic larvae and spores of many marine fishes, invertebrates, and macroalgaes (Shanks et al 2003). The distances traveled by these dispersive entities, or “propagules,” range from a few meters to several hundred kilometers (Aylor et al 1982; Aylor 2003; Nathan et al 2008; Shanks et al 2003; Shanks 2009; Suter 1999). Because adults of these species are usually sedentary or sessile by comparison (Levin et al 2003), understanding how these propagules disperse is critical for predicting the spatial and temporal dynamics of adult populations.

The propagules of many organisms are passive, at the mercy of the media in which they disperse (Okubo and Levin 2001). However, the propagules of some animals exhibit surprising control over their fates through behavior. The larvae of several coastal marine invertebrates, for example, regulate their depths during dispersal to exploit vertical variations in currents, food abundance, and predation risk (Largier 2003; Levin 2006; Morgan 1995; Shanks 1995, 1986). Larval active swimming speeds are typically less than 2 cm s^-1^ (Chia et al 1984), but conditions in the water column vary on length scales of 1 to 100 m (Cowen et al 2000; Morgan 1995; Shanks 1995; Sherr et al 2005). Thus, swimming for just a few hours can move larvae from food-poor depths to food-rich ones, or from offshore currents to onshore ones. These cross-shore currents usually flow faster than larvae can swim (1 to 30 cm s^-1^), so vertical swimming also provides an efficient mechanism by which larvae can regulate their cross-shore movement (Shanks 1995). Therefore, despite their poor locomotive abilities, larvae dramatically alter their dispersal outcomes through interactions with the structure of their environment.

In principle, many of the pressures that shape the behaviors of coastal invertebrate larvae are easily understood, making them ideal subjects for exploring how propagule behavior influences dispersal. Larvae are spawned in vast quantities from nearshore habitats (Gerber et al 2014; Rumrill 1990), and develop in the water column for a species-specific period of time called the larval duration (Levin and Bridges 1995). They are simultaneously transported off- and alongshore by ocean currents, enabling dispersal between coastal habitats (Shanks et al 2003; Shanks 2009, 1995) and an escape from nearshore predators (Morgan 1995; Pechenik 1999). Some larvae feed during dispersal, while others, supplied with a maternal energy source, do not (Levin and Bridges 1995). However, all larvae must settle into nearshore habitats at the end of the larval duration and perform a costly metamorphosis to their post-larval forms (Elkin and Marshall 2007; Pechenik 1999; Shanks 1995). This is a perilous journey during which most larvae succumb to predation and starvation or are lost offshore (Morgan 1995; Rumrill 1990). Larval behaviors are, at least in the short term, driven by the requirements that individuals return to shore, avoid predation and starvation, and reserve energy for metamorphosis (Morgan 1995; Shanks 1995). It is less clear how behaviors might be shaped by the long-term benefits of dispersal between populations.

**Fig. 1:**
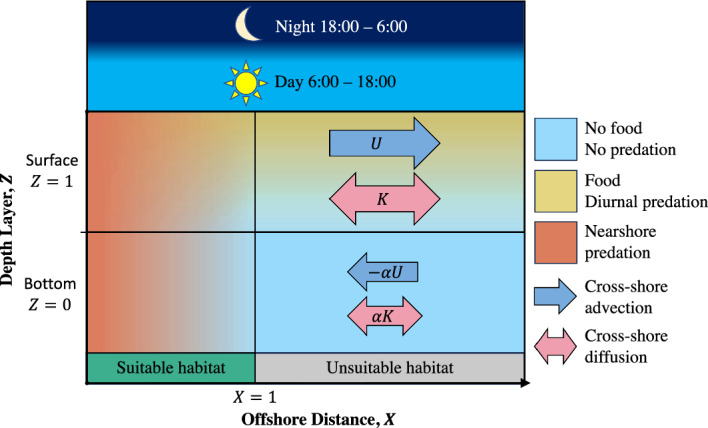
Schematic diagram of the modeled coastal environment and day-night cycle. The environment featured surface and bottom depth layers, $Z = 1,0$ (respectively), and a nearshore habitat suitable for settling and metamorphosis, $X \le 1$. To approximate the flow pattern typical of coastal upwelling, we assumed the surface layer moved offshore with velocity *U* d^-1^ and diffusivity *K* d^-1^ while the bottom layer moved onshore with reduced velocity $\alpha U$ d^-1^ and diffusivity $\alpha K$ d^-1^. In the diurnal predation scheme, the surface featured elevated mortality during daylight hours. In the nearshore predation scheme, both depth layers featured elevated mortality over the nearshore habitat. In both schemes, the surface featured abundant food

Behaviors observed in the field and laboratory seem to reflect predictable elements of the environment in which dispersal occurs, as well as features of larvae themselves. Diel vertical migrations (DVM), in which larvae visit the surface at night and descend to the bottom during the day, are frequently reported by laboratory and field studies of species with feeding larvae, such as the crabs *Atelecyclus rotundus* (dos Santos et al 2008), *Carcinus maenas* (Queiroga et al 2007), and *Cancer* spp. (Shanks 1986). This behavior allows larvae to exploit abundant food near the surface while escaping visually guided predators during the photoperiod. Another common behavior is ontogenetic vertical migration (OVM), in which larvae vary their depths throughout development according to their changing needs over time. For example, larvae of the barnacle *Balanus nubilus* are only able to feed during the first part of dispersal, so they begin in the food-rich surface layer and then migrate to the food-poor bottom (Tapia et al 2010). In an upwelling circulation featuring an offshore-moving surface layer atop an onshore-moving bottom layer (Fig. 1A), this behavior may also facilitate transport away from nearshore hazards at the start of dispersal and delivery toward nearshore habitats at the end (Shanks 1995).

While these qualitative arguments suggest that vertical swimming behaviors benefit invertebrate larvae in specific ways (e.g., delivery toward shore or predator avoidance), it is not obvious how these behaviors affect overall fitness. For instance, because food sources and predators commonly co-occur near the water’s surface (Chavez and Messié 2009; Lampert 1989), it is unclear how larvae should swim to simultaneously balance onshore delivery with predator avoidance and energy needs. Furthermore, variability of current regimes, food and predator abundance, and other factors over time and space suggest that behaviors may be adaptive in a wide range of conditions. It is often difficult to predict how the performance of a larval swimming behavior is affected by these variables *per se* or in combination. Consequently, mathematical models are frequently used to explore how larval swimming might affect the dispersal of larvae between coastal habitats (Cowen et al 2006; Marta-Almeida et al 2006; Meyer et al 2021; Owens and Rothlisberg 1991; Paris et al 2007; Rothlisberg et al 1983; Sundelöf and Jonsson 2012). These studies support the hypothesis that observed vertical swimming patterns result in nearshore retention of larvae in realistic habitats, but consider only a few prescribed behaviors (e.g., diel vertical migrations) and are rarely validated by data. James et al (2019) instead attempted to construct behaviors that could recreate vertical distributions of larvae measured in the field, assuming only that larvae change their swimming velocity at key moments throughout the tidal cycle. The authors found that some, but not all, observed distributions could be reproduced by such behaviors, underscoring the importance of considering more than one type of swimming behavior and validating the results of dispersal models.

Few of the studies listed above considers the effects of vertical swimming behaviors upon mortality risk and energy use. Meyer et al (2021) addressed this gap by modeling how larval delivery, predation risk, energy use, and food access are concurrently affected by a broad class of prescribed swimming behaviors, including diel vertical migrations and ontogenetic depth changes. The authors showed that while some behaviors successfully retain larvae nearshore and others improve feeding opportunities, no behaviors considered were able to do both simultaneously in the idealized environment of their model. In other words, remaining nearshore during development and acquiring energy for metamorphosis, escaping nearshore predators, and dispersing between habitats were conflicting needs. This theoretical result raises intriguing questions: how should larvae swim in order to balance these requirements? And, given the wide range of behaviors larvae could exhibit, why do behavioral archetypes like diel vertical migrations appear to be so common in nature?

In this paper, we addressed these questions using a simple mathematical model that described the cross-shore movement, vertical swimming, and energy content of a single larva. We did not assume any specific type of larval vertical swimming behavior. Instead, we used dynamic programming, an optimal control method frequently applied in behavioral ecology (Mangel and Clark 1988), to construct behaviors *de novo* that maximized a performance metric combining the benefits of onshore delivery, predator avoidance, and energy conservation. For simplicity, we limited this analysis to the case of larvae dispersing in a coastal environment characterized by upwelling circulation. Although many other ecologically important flow regimes exist, upwelling occurs in many ecosystems in which larval locomotion has been well studied (Meyer et al 2021), such as off the west coasts of the United States, Chile, Portugal, and South Africa (Chavez and Messié 2009). We compared the resulting optimal behaviors against passive drifting and three swimming behaviors commonly reported in the literature: diel vertical migrations, a single ontogenetic vertical migration, and combinations thereof. Our results underscored the importance of carefully including propagule behavior in discussions and models of dispersal, especially when making predictions on ecological and evolutionary time scales.

## Methods

We developed a mathematical model that estimated the effect of a larva’s swimming behavior upon its ability to survive dispersal, locate a suitable habitat, and reserve energy for metamorphosis. This model was based on that of Meyer et al (2021), which simulated the dispersal of an individual larva over time in a simplified coastal environment approximating an upwelling regime (Fig. 1). We altered this model in three key ways. First, we made the model more amenable to optimization via dynamic programming by using a discrete time variable, rather than a continuous one (Mangel and Clark 1988). Second, we added a state variable to the model that captured the larva’s energy content over time. Third, we derived a “Trajectory Score” that allowed us to compare how different vertical swimming behaviors affected individuals’ chances of surviving through metamorphosis. Optimal swimming behaviors maximized the expected value of the Trajectory Score. However, the Trajectory Score was also useful for comparing optimal and prescribed (non-optimal) swimming behaviors in various scenarios.

### Model Description

#### Notation

Let the coordinates *X*, *Y*, and *Z* denote the cross-shore position, alongshore position, and depth (respectively) of a point in the coastal environment, and let *E* denote an amount of energy. With subscripts *t*, these variables represent the coordinates $X_t, Y_t, Z_t$ and energy reserve $E_t$ of a modeled larva *t* days after spawning. Bolded variables $\pmb X,\pmb Y,\pmb Z,\pmb E$ represent vectors of all such values over time; for example,

$$\begin{aligned} \pmb {X} = \{X_0,X_{\Delta t},X_{2\Delta t},\ldots \}. \end{aligned}$$

#### Environment

The environment considered by our model consisted of a long, homogeneous coastline located at $X = 0$, a coastal environment characterized by $X > 0$, and two depth layers: a bottom layer, $Z = 0$, and a surface layer, $Z = 1$ (Fig. 1). Due to our focus on how vertical swimming affected larval movement toward and away from the coast, rather than parallel to it, we did not explicitly model this environment’s alongshore dimension, *Y*. Coastal invertebrates vary in their typical larval dispersal distances and post-larval habitat sizes (Shanks 2009; Shanks et al 2003). We avoided tailoring our model to the biology of any particular species by scaling the offshore distance variable *X* with respect to size of the post-larval habitat. By scaling distances in this way, we assumed that the post-larval habitat occupied a nearshore strip of width 1 along the coastline, $X \in [0,1]$. All references to distances, velocities, and diffusion rates should be interpreted relative to the actual width (e.g., in meters) of this habitat.

#### Movement

We modeled an individual larva of a hypothetical invertebrate species with a larval duration of *T* days. We focused our analysis on species with a medium larval duration of $T = 20$ days, but also considered shorter and longer durations of $T = 6$ and 80 days in Supplement 3 (Meyer et al 2021; Shanks 2009; Shanks et al 2003). At $t = 0$, the modeled larva spawned into the bottom depth layer from a random location within the post-larval habitat. It aimed to settle back into this habitat at the end of dispersal, $t = T$. Between spawning and settling, the larva changed its vertical position by swimming— that is, through a sequence of depth changes

1 $$\begin{aligned} \pmb {\Delta Z} = \{\Delta Z_0,\Delta Z_{\Delta t},\ldots ,\Delta Z_{T - \Delta t},\Delta Z_T\}. \end{aligned}$$

These depth changes took the values $\Delta Z = +1$ for swimming from the bottom layer to the surface, $\Delta Z = -1$ for swimming from the surface layer to the bottom, and $\Delta Z = 0$ for remaining in the same layer. The time-step $\Delta t = 1/8$ days was slightly shorter than our estimate of the time required for a single vertical migration, but was suitably short for approximating cross-shore diffusion as a discrete-time process (see Supplement 1.2). While in layer *Z*, the modeled larva was subjected to a cross-shore current with mean velocity (advection) $U_Z$ d^-1^ and variance (eddy diffusivity) $K_Z$ d^-1^. Altogether, the modeled larva’s position changed throughout dispersal according to the equations

2 $$\begin{aligned}{} & {} X_0 \sim \text {Uniform}([0,1]), \end{aligned}$$

3 $$\begin{aligned}{} & {} Z_0 = 0, \end{aligned}$$

4 $$\begin{aligned}{} & {} X_{t + \Delta t} = X_t + U_{Z_t}\Delta t + \xi _t \sqrt{2K_{Z_t}\Delta t} , \end{aligned}$$

5 $$\begin{aligned}{} & {} Z_{t + \Delta t} = Z_t + \Delta Z_t \end{aligned}$$

for $t = 0,\Delta t,\ldots ,T-\Delta t$, where $\xi _t$ are independent standard normal random variables. To model scenarios ranging from still water to upwelling circulation, we assumed that the surface layer featured an offshore current, $U_1 = U \ge 0$, while the bottom layer featured a slower, compensatory onshore current, $U_0 = -\alpha U$. The constant $\alpha \in [0,1]$ roughly represented the ratio of the depths of the surface and bottom layers. Similarly, we assumed that the surface layer featured stronger eddy diffusion than the lower layer, $K_1 = K > 0$ and $K_0 = \alpha K$. We referred to scenarios with $U = 0$ as “still water” due to the lack of directed currents; in these cases, the cross-shore movement of larvae was entirely driven by random diffusion.

#### Energetics

We aimed to model the energy reserve of the larva as simply as possible while capturing constraints imposed by food abundance, maintenance, growth, and locomotion. Let $E_t$ denote the the size of the modeled larva’s energy reserve at time *t*. We rescaled $E_t$ and related quantities by the amount of energy needed for a complication-free metamorphosis, which varies across species (Bennett and Marshall 2005; Lucas et al 1979; Rodriguez et al 1990; Thiyagarajan et al 2003; Videla et al 1998). Consequently, the modeled larva aimed to finish dispersal with at least $E_T = 1$ energy unit.

During dispersal, the modeled larva expended energy on maintenance and growth at rate *G* d^-1^. We assumed that the larva spawned with enough energy for maintenance throughout dispersal, *GT*, plus a deficit or maternally supplied surplus of size *S*. We focused our analysis on feeding larvae with no surplus, $S = 0$, such that all energy for movement and metamorphosis needed to be acquired during dispersal. Nonfeeding larvae with $S > 0$ and feeding larvae with deficits $S < 0$ are considered in Supplement 4. Due to our interest in cases where food is concentrated in the surface, we assumed that the modeled feeding larva obtained energy at rate *FZ* d^-1^ (that is, rates *F* d^-1^ in the surface layer and 0 d^-1^ in the bottom). Throughout this paper, we used *F* as a proxy for food abundance, although technically *F* is the product of food abundance and the larval feeding rate. Finally, we assumed that the larva was neutrally buoyant and regulated its depth by actively swimming upward or downward, rather than passively rising or sinking. Maintaining a constant depth cost 0 energy units, while each vertical migration in either direction cost $V > 0$ units.

**Fig. 2:**
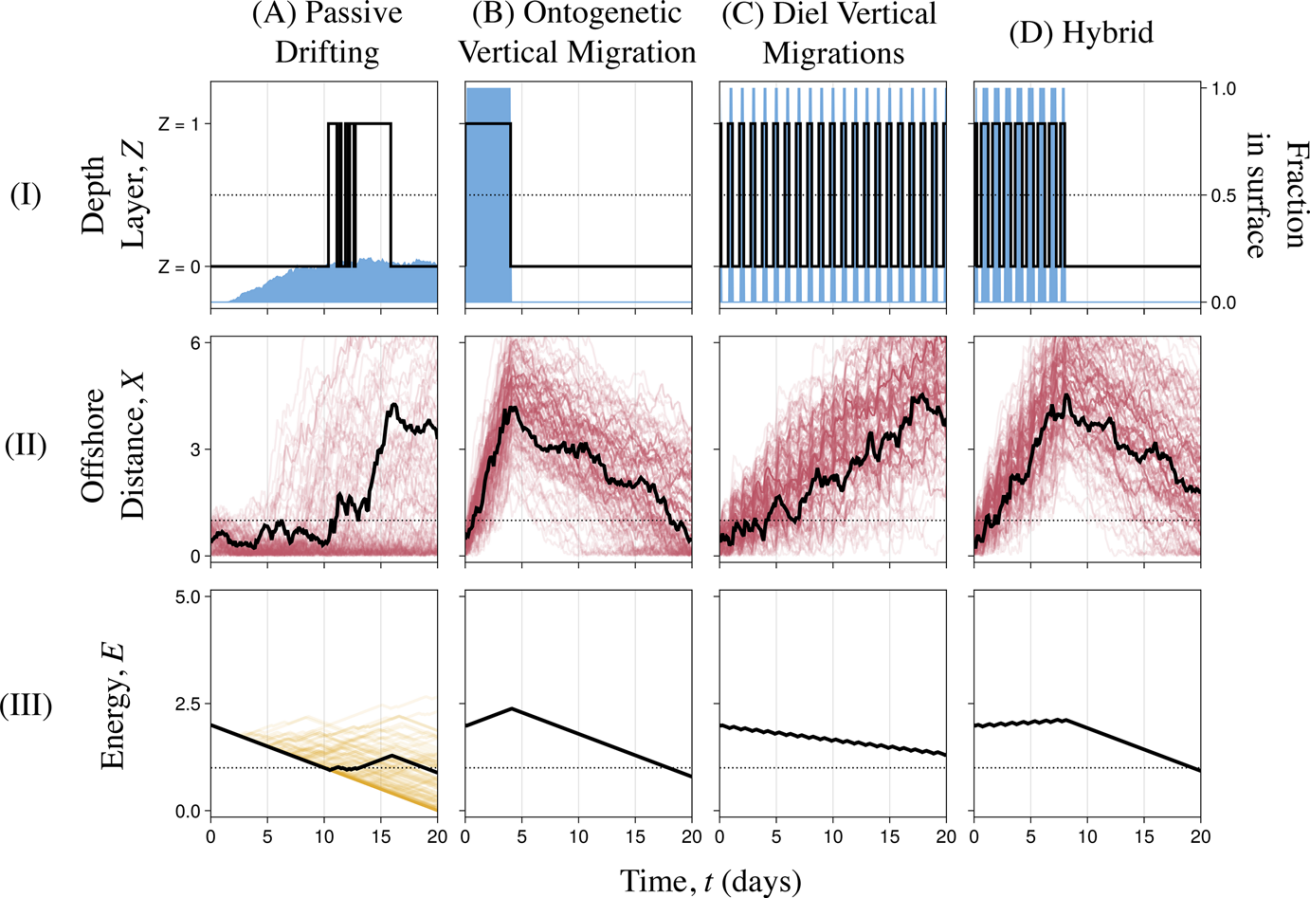
The four non-optimal vertical swimming behavioral archetypes. **A** Passive Movement, **B** Ontongenetic Vertical Migration (OVM), **C** Diel Vertical Migrations (DVM), **D** Hybrid. Row I shows the fraction of simulated larvae in the surface over time (blue, right axis) and a single simulation of *Z*(*t*) for each behavior (black lines, left axis). Recall that *Z*(*t*) is stochastic for Passive Movement but deterministic for the other behaviors. Rows II and III show *X*(*t*) and *E*(*t*), respectively, for 100 simulated larvae performing each behavior. The black lines in Rows II-III show examples *X*(*t*) and *E*(*t*) for the same simulated larvae highlighted in Row I (Color figure online)

**Fig. 3:**
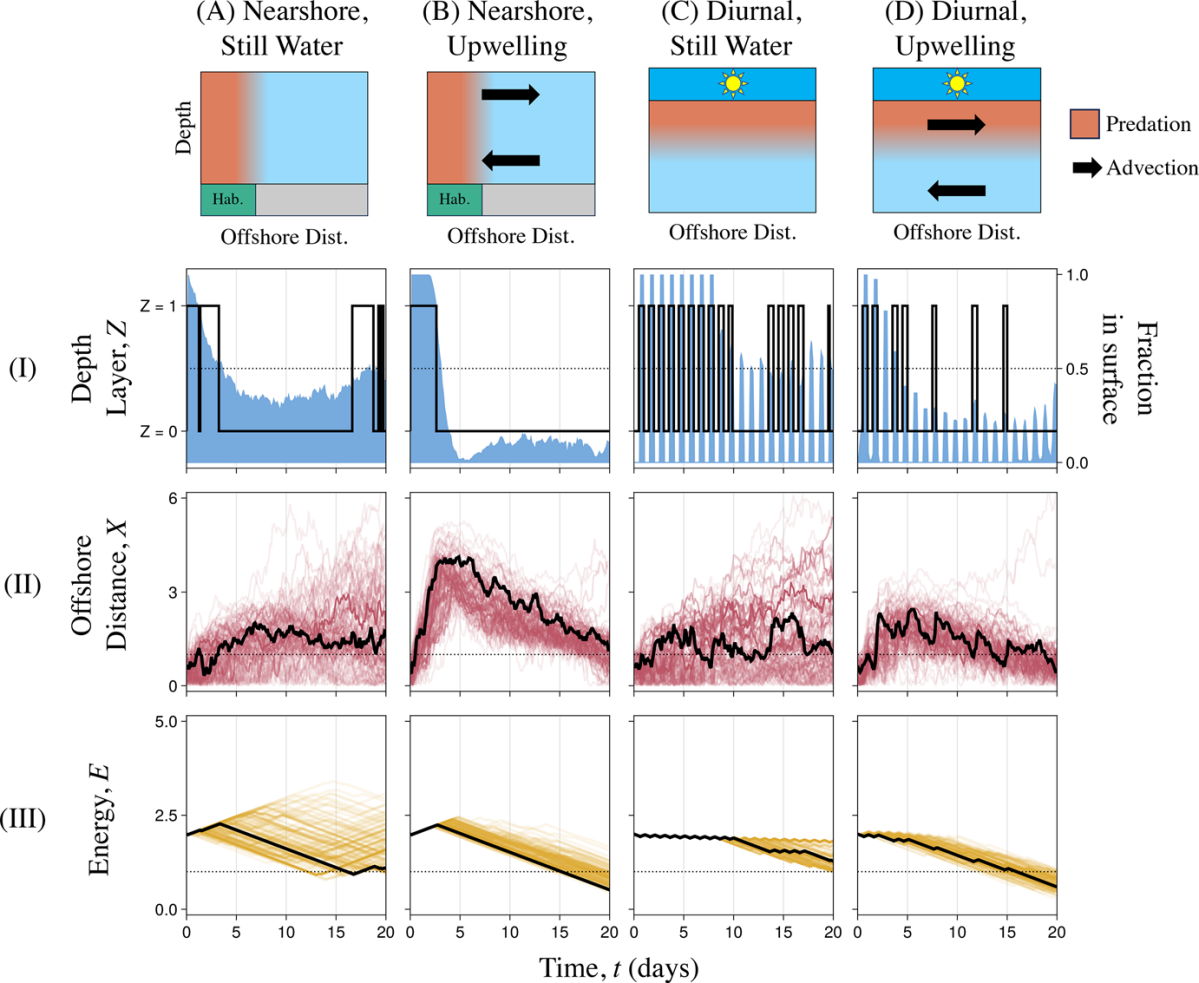
Optimized trajectories of simulated larvae subject to **A** nearshore predation in still water, **B** nearshore predation with upwelling, **C** diurnal predation in still water, and **D** diurnal predation with upwelling. Diagrams in the top row illustrate these environmental conditions. Within each column, the solid black curves show the (I) depth, $Z_t$, (II) offshore distance, $X_t$, and (III) energy reserve, $E_t$, of a typical simulated larva under each set of conditions. As in Fig. 2, the blue shading in Row I shows the fraction of several optimized larvae in the surface over time (right axis), while the red and yellow curves in Rows II and III each show 100 additional optimized trajectories $X_t$ and $E_t$, respectively (Color figure online)

The modeled larva was unable to carry more than $E_\textrm{max}$ energy units at any instant and experienced starvation whenever $E_t = 0$; otherwise, we asserted that $0< E_t < E_\textrm{max}$. These restrictions precluded biologically unrealistic scenarios, but were rarely invoked. Limiting $E_t$ to $E_\textrm{max}$ did not prevent simulated larvae from gathering sufficient energy for metamorphosis, so it is unlikely that the choice of $E_\textrm{max}$ affected our results (Fig. 3 and Supplement 3). Because the extent to which larvae actually experience starvation in nature is uncertain (Morgan 1995), we chose model parameters that made starvation unlikely for a feeding larva with larval duration of $T = 20$ days (Fig. 2, row III). However, starvation did not occur during optimized simulations in any other scenario we considered.

Combining the assumption above, the larva’s energy reserve changed according to the equations

6 $$\begin{aligned}{} & {} E_0 = S + GT, \end{aligned}$$

7 $$\begin{aligned}{} & {} E_t' = E_t + (FZ-G)\Delta t - V|\Delta Z|, \end{aligned}$$

8 $$\begin{aligned}{} & {} E_{t+\Delta t} = {\left\{ \begin{array}{ll} 0 &{}\hbox {if } E_t' \le 0, \\ E_t ' &{}\hbox {if } 0< E_t' < E_\textrm{max}, \\ E_\textrm{max} &{}\hbox {if } E_t' \ge E_\textrm{max} \end{array}\right. } \end{aligned}$$

for $$t = 0,\ldots ,T - \Delta t$$.

### Vertical Swimming Behavioral Archetypes

The main goal of this study was to compute optimal vertical swimming behaviors for larvae under various circumstances, and then to compare those against behavioral “archetypes” that appear frequently in the literature. The four archetypes we chose are detailed below.

**Passive Drifting.** As a null reference, we considered a passive drifting archetype in which vertical movement was due to diffusion only (Fig. 2A.I). A full description of this behavior is presented in Supplement 2.

**Ontogenetic Vertical Migration.** Larvae of some invertebrate species, such as the barnacle *Balanus nubilus*, reside near the water’s surface for the first portion of dispersal before performing a single ontogenetic vertical migration (OVM) from the surface into the bottom (Tapia et al 2010). We considered an OVM-like behavioral archetype in which the modeled larva with larval duration $T = 20$ days spent four days in the surface layer before migrating to the bottom layer for the remaining 16 days of dispersal (Fig. 2B.I).

**Diel Vertical Migration Archetype.** Diel vertical migration (DVM), in which larvae visit the surface during darkness and return to the bottom during daylight, is among the most widely documented larval swimming behaviors (Meyer et al 2021). We considered a DVM-like behavioral archetype in which larvae visited the surface each night from 21:00 until 03:00 (Fig. 2C.I). Past modeling studies have demonstrated that small details of how DVM is modeled can profoundly affect predictions of larval dispersal success (Meyer et al 2021; Sundelöf and Jonsson 2012). Thus, we considered alternate DVM archetypes in Supplement 2.

**Hybrid Archetype.** Larvae of some species, such as the brittle star *Ophiocomina nigra*, exhibit behaviors in between DVM and OVM, visiting the surface nocturnally for the first part of dispersal before residing continually in the bottom (Guillam et al 2020). We considered a Hybrid behavioral archetype in which the modeled larva with larval duration $T = 20$ days visited the surface layer each evening from 18:00 until 06:00 for the first 8 days of dispersal, then migrated to the bottom layer for the remaining 12 days of dispersal (Fig. 2D.I).

### The Trajectory Score

Each vertical swimming behavior was represented as a sequence of depth changes, $\Delta Z$. Given the stochasticity of larval dispersal in nature (and as modeled), each behavior resulted in many possible larval trajectories, $(\pmb {X},\pmb {Z},\pmb {E})$. Each such trajectory exposed larvae to potentially the same conditions, although individuals on the same trajectory reached different ultimate fates (e.g., death during dispersal versus settling near the shore). We derived a Trajectory Score, *J*, to measure how well the trajectories resulting from different behaviors allowed larvae to balance four conflicting needs: avoiding predators, avoiding starvation, settling into the nearshore habitat, and reserving energy for metamorphosis.

The Trajectory Score was based on the probability of a larva surviving a trajectory through metamorphosis, rather than the experience of any individual larva. We began with the expression

9 $$\begin{aligned} \widetilde{J}[\pmb {X},\pmb {Z},\pmb {E}] = \log \Pr \big (\hbox {survive through metamorphosis } |\ \pmb {X},\pmb {Z},\pmb {E}\big ) + C, \end{aligned}$$

where *C* was an arbitrary constant for absorbing terms that did not depend on the trajectory. We assumed that the probability in (9) could be written as

10 $$\begin{aligned} \begin{aligned}&\Pr \big (\hbox {survive through metamorphosis } |\ \pmb {X},\pmb {Z},\pmb {E}\big ) \\&= \Pr \big (\hbox {survive dispersal}\ \big |\ \pmb {X},\pmb {Z},\pmb {E}\big ) \cdot \Pr \big (\hbox {metamorphose}\ \big |\ \hbox { survive dispersal on}\ \pmb {X},\pmb {Z},\pmb {E} \big ). \end{aligned} \end{aligned}$$

During dispersal, we assumed that larvae following trajectory $(\pmb {X},\pmb {Z},\pmb {E})$ experienced time- and location-dependent predation rate $\mu _\textrm{pred}(t,X,Z)$ and starvation-induced mortality rate $\mu _\textrm{starve}(E)$, such that

11 $$\begin{aligned} \Pr \big (\hbox {survive dispersal}\ \big |\ \pmb {X},\pmb {Z},\pmb {E}\big ) = \exp \left[ -\sum _t \Big (\mu _\textrm{pred}(t,X_t,Z_t) + \mu _\textrm{starve}(E_t)\Big ) \Delta t\right] .\nonumber \\ \end{aligned}$$

Whether larvae successfully metamorphosed at the end of this dispersal trajectory depended on their final offshore position, $X_T$, and energy reserve, $E_T$, with the assumed form

12 $$\begin{aligned} \Pr \big (\hbox {metamorphose}\ \big |\ \hbox {survive dispersal on}\, \pmb {X},\pmb {Z},\pmb {E} \big )\nonumber \\ = \exp \Big [-a_\textrm{settle} \phi _\textrm{settle}(X_T) -a_\textrm{meta} \phi _\textrm{meta}(E_T)\Big ] \end{aligned}$$

Substituting these probabilities into the expression for $\widetilde{J}$ resulted in a preliminary trajectory score

13 $$\begin{aligned} \widetilde{J}= & {} \underbrace{-\sum _t \mu _\textrm{pred}(t,X_t,E_t)\Delta t}_{\widetilde{J}_\textrm{pred}}\ +\ \underbrace{-\sum _t \mu _\textrm{starve}(E_t)\Delta t}_{\widetilde{J}_\textrm{starve}} \\{} & {} - a_\textrm{settle}\phi _\textrm{settle}(X_T) \nonumber - a_\textrm{meta}\phi _\textrm{meta}(E_T) + C \end{aligned}$$

with negative penalty terms representing the four conflicting needs listed above. To our knowledge, none of the functions $\mu _\textrm{pred}$, $\mu _\textrm{starve}$, $\phi _\textrm{settle}$, and $\phi _\textrm{meta}$ have been empirically measured in a way that applies to multiple species. Below, we describe simplistic choices for these functions that capture the relevant biology, and combine unknown variables to obtain a final expression for *J* as the sum of four non-negative Sub-Scores,

14 $$\begin{aligned} J[\pmb X,\pmb Z, \pmb E] = p_\textrm{pred} J_\textrm{pred} + {p_\textrm{starve} J_\textrm{starve}} + p_\textrm{settle} J_\textrm{settle} + p_\textrm{meta} J_\textrm{meta}, \end{aligned}$$

with non-negative weights $p_i$ that sum to 1.

#### Components of the Trajectory Score

**Predation Schemes and the Predation Score.** We considered two predation schemes in our analysis. In the *nearshore predation scheme*, larvae experienced increased predation over the nearshore region $X \in [0,1]$ than offshore, $X > 1$. In the *diurnal predation scheme*, larvae experienced increased predation in the surface, $Z = 1$, during daylight, which we assumed to last from 6:00 to 18:00 each day. The nearshore scheme was inspired by the hypothesis that planktonic development is advantageous because it removes vulnerable larvae from nearshore predators, while the diurnal scheme was inspired by the hypothesis that DVM-like behaviors were selected for through light-guided predation (Burgess et al 2016; Levin 2006; Pechenik 1999; Morgan 1995; Strathmann 1974). We considered these schemes separately because their relative contributions to mortality during dispersal are not well-understood.

Each scheme defined a high-predation “region” within the coastal environment: $X \in [0,1]$ in the nearshore scheme, and $Z = 1$ with $t \mod 1 \in [0.25,0.75]$ (the daylight period) in the diurnal scheme. We assumed that larvae died of predation at rate $\mu _\textrm{pred}^0$ outside of these regions and at rate $\mu _\textrm{pred}^0 + \mu _\textrm{pred}^1$ inside of these regions. This resulted in

15 $$\begin{aligned} \begin{aligned} \widetilde{J}_\textrm{pred} + C&= -\mu _\textrm{pred}^1[\hbox {days inside high-predation region}] -\mu _\textrm{pred}^0 T + C \\&= \mu _\textrm{pred}^1[\hbox {days outside high-predation region}] - (\mu _\textrm{pred}^0 + \mu _\textrm{pred}^1) T + C \\&= \underbrace{\mu _\textrm{pred}^1 T^{-1}}_{a_\textrm{pred}}\ \cdot \ \underbrace{[\hbox {fraction of larval duration outside high-predation region}]}_{J_\textrm{pred}}\ +\ C. \end{aligned} \end{aligned}$$

Here and elsewhere, the constant *C* was redefined as needed.

**Starvation Score.** We assumed that modeled larvae were unaffected by starvation unless $E_t = 0$, in which case they experienced starvation-induced mortality at rate $\mu _\textrm{starve}^0 > 0$. This resulted in

16 $$\begin{aligned} \begin{aligned} \widetilde{J}_\textrm{starve} + C&= -\mu _\textrm{starve}^0 [\hbox {days with }E_t = 0] + C \\&=\mu _\textrm{starve}^0 [\hbox {days with }E_t> 0] - \mu _\textrm{starve}^0 T + C \\&= \underbrace{\mu _\textrm{starve}^0 T^{-1}}_{a_\textrm{starve}}\ \cdot \ \underbrace{[\hbox {fraction of larval duration with }E_t > 0]}_{J_\textrm{starve}}\ +\ C. \end{aligned} \end{aligned}$$

**Settling and Metamorphosis Scores.** We chose $\phi _\textrm{settle}$ to penalize only larval trajectories ending beyond the nearshore habitat; that is, $\phi _\textrm{settle}(X_T) > 0$ if and only if $X_T > 1$:

17 $$\begin{aligned} \phi _\textrm{settle}(X_T) = {\left\{ \begin{array}{ll} 0 &{}\hbox {if } X_T \in [0,1], \\ 1 - X_T^{-1} &{}\hbox {if } X_T > 1. \end{array}\right. } \end{aligned}$$

Similarly, we chose $\phi _\textrm{meta}$ to penalize trajectories ending with insufficient energy for a complication-free metamorphosis,

18 $$\begin{aligned} \phi _\textrm{meta}(E_T) = {\left\{ \begin{array}{ll} 0 &{}\hbox {if } E_T \ge 1, \\ 1 - E_T &{}\hbox {if } E_T < 1. \end{array}\right. } \end{aligned}$$

We rewrote the third and fourth terms of equation (14) as follows, defining non-negative Settling and Metamorphosis Scores:

19 $$\begin{aligned} -a_i\phi _i + C = a_i \underbrace{(1 - \phi _i)}_{J_i} + C \end{aligned}$$

for $$i = \textrm{settle},\textrm{meta}$$.

**Normalization and Weights.** Substituting (15), (16), and (19) into the expression for $\widetilde{J}$ resulted in

20 $$\begin{aligned} {\widetilde{J}}[\pmb X,\pmb Z, \pmb E] = a_\textrm{pred} J_\textrm{pred} + a_\textrm{starve} J_\textrm{starve} + a_\textrm{settle} J_\textrm{settle} + a_\textrm{meta} J_\textrm{meta} + C. \end{aligned}$$

Dropping the constant *C*, dividing by $\sum _i a_i$, and defining weights $p_i = a_i / \sum _j a_j$ resulted in the Trajectory Score *J* in equation (14). The weights $p_i$ represented the intensity of predation, stringency of energetic requirements, and habitat specificity of the species of interest. To avoid skewing our results toward any single need at the cost of the others, we set all four weights to $p_i = 0.25$. This choice was justified in Supplement 5, where we showed that the optimal swimming behaviors computed using these and other weights differed only slightly.

#### Interpreting the Trajectory Score

The Trajectory Score *J* and each subscore $J_i$ took values between 0 and 1. Greater values of $J_i$ indicated that a trajectory better allowed simulated larvae to meet need *i*. Values of *J* close to 1 indicated that a trajectory allowed larvae to simultaneously avoid predation and starvation, settle close to shore, and reserve energy for metamorphosis.

Before proceeding with our analysis, some subtle points about the model and the Trajectory Score must be made explicit. The model simulates larval trajectories, rather than the fates of individual larvae. Real individuals may die during dispersal. Our model does not simulate trajectories leading to death, but instead simulates full trajectories and then computes (using the Trajectory Score) the probability of death having occurred along them. To this point, the Trajectory Score should be viewed as a property of a simulated trajectory or a population of larvae following that trajectory, and not of an individual simulated larva. For example, a trajectory that exposes larvae to intense predation but ends in ideal settling conditions may receive a good Trajectory Score. This indicates that among a population of larvae following the same trajectory, the loss of larvae during dispersal is made up for by the good conditions encountered by survivors, resulting in a relatively high fraction of larvae ultimately completing metamorphosis. It does not mean that an individual larva can compensate for dying during dispersal if it was headed toward ideal conditions.

### Optimal Vertical Swimming Behaviors

Each swimming behavior $\pmb {\Delta Z}$ resulted in many possible larval trajectories $(\pmb {X},\pmb {Z},\pmb {E})$. We computed optimal behaviors that maximized the expected Trajectory Score across those trajectories:

21 $$\begin{aligned} {\mathop {{{\,\textrm{maximize}\,}}}\limits _{\pmb {\Delta Z}}}\ \textbf{E}\left( J[\pmb {X},\pmb {Z},\pmb {E}]\ \Big |\ \pmb {\Delta Z}\right) \hbox { subject to dynamics } (2)-(8). \end{aligned}$$

This stochastic, discrete-time optimal control problem was readily solved using the method of dynamic programming. In each scenario we considered, dynamic programming constructed an optimal swimming “policy,” or a sequence of functions $\Delta Z_t(X,Z,E)$ for choosing the best depth change for a larva in state (*X*, *Z*, *E*) at time *t*, rather than proposing a single best behavior. These policies were idealizations that assumed larvae were aware of and able to respond to their present and anticipated future states. Ecologically motivated introductions to optimal control and dynamic programming are available in Clark (1990) and Mangel and Clark (1988).

## Results

Our main analysis consisted of three parts. First, we examined the larval trajectories produced by the passive drifting and the Ontogenetic Vertical Migration (OVM), Diel Vertical Migrations (DVM), and Hybrid behavioral archetypes over a larval duration of $T = 20$ days. Second, we explored the larval trajectories produced by optimally swimming larvae under several environmental and biological conditions. Finally, we used the Trajectory Score to directly compare the behavioral archetypes against the optima. We investigated how each behavior’s Trajectory Score depended on current strength and food abundance, and then determined which behaviors were most advantageous with respect to predator and starvation avoidance, delivering larvae to shore, and reserving energy for metamorphosis.

### The Behavioral Archetypes

Visualizing the trajectories produced by passive drifting and the three behavioral archetypes helped us establish expectations for what optimal behaviors could look like. For each of these behaviors, we simulated several larval trajectories using the default biological and environmental conditions described in Table 1: feeding larvae with a 20-day larval duration subjected to upwelling and limited food abundance. Passive drifting generally produced larval trajectories with few short visits to the surface (Fig. 2A.I). Given the surface layer’s food content and offshore current, this resulted in many simulated larval trajectories remaining close to shore throughout dispersal (Fig. 2A.II) but failing to gather energy for metamorphosis (Fig. 2A.III). No simulated trajectories resulted in starvation due to the initial condition $E_0 = GT$. Nonetheless, the Passive behavior performed poorly, suggesting that optimal behaviors will include active and deliberately timed vertical migrations.

**Table 1:** Summary of symbols used in the formulation of the larval swimming model

Symbol	Meaning	Value(s)
*t*	Time since spawning (days)	0 to *T*
$\Delta t$	Time-step of model	0.125
*T*	Larval duration (days) *Medium (default)*	20
	Short*	6
	Long*	80
$X_t$	Larva’s offshore distance at time *t*	Non-negative
$Z_t$	Larva’s depth-layer at time *t*	0 or 1
$\Delta Z_t$	Larva’s depth change immediately after time *t*	-1, 0, or 1
*U*	Offshore velocity of surface layer *Upwelling (default)*	1
	*Still water*	0
*K*	Cross-shore eddy diffusivity of the surface layer	0.2
$\alpha$	Ratio of current strengths between bottom and surface layers	0.25
$E_t$	Larva’s energy reserve at time *t*	0 to $E_\text {max}$
$E_\text {max}$	Maximum energy reserve size of a larva	5
*S*	Size of initial energy surplus or deficit *Feeding with no surplus (default)*	0
	Feeding with 80-day larval duration*	$-6$
	Feeding with 20-day larval duration and deficit*	$-1$
	Nonfeeding with surplus*	1 or 3
*G*	Rate of energy use for maintenance and growth	0.1
*F*	Rate of energy intake in the surface layer *Low food (default)*	0.2
	High food*	0.5
	Nonfeeding*	0
*V*	Energetic cost of vertical migration in either direction	0.004
*J*	Trajectory Score of a larval trajectory	0 to 1
$$\begin{array}{c} J_\textrm{pred}, J_\textrm{starve}, \\ J_\textrm{settle}, J_\textrm{meta} \end{array}$$	Predation avoidance, starvation avoidance, settling site, metamorphosis sub-scores	0 to 1
$$\begin{array}{c} p_\textrm{pred}, p_\textrm{starve}, \\ p_\textrm{settle}, p_\textrm{meta} \end{array}$$	Predation avoidance, starvation avoidance, settling site, metamorphosis weights	0.25

All distance-related parameters (e.g., current velocities) and energetic parameters are relative to the width of the nearshore habitat and the cost of metamorphosis, respectively. Explanations of these default values are presented in Supplement 1. Variables and scenarios marked with asterisks, including different values for the weights $p_i$, are explored in the Supplement, but not the main text

Unlike the Passive archetype, the OVM, DVM, and Hybrid archetypes followed prescribed depth changes over time. This resulted in deterministic energy accumulation over time (Figs. 2B–D.III), although cross-shore movement remained stochastic due to diffusion (Figs. 2B–D.II). The OVM and Hybrid archetypes resulted in larval trajectories that fell short of the $E_T = 1$ energy units required for complication-free metamorphosis, but still ended near or in the nearshore habitat, $X_T \in [0,1]$. In contrast, the DVM archetype resulted trajectories that always ended with enough energy, but frequently delivered larvae too far offshore. This suggested, as indicated by Meyer et al (2021), that larvae performing these behaviors in upwelling are unlikely to simultaneously gather sufficient energy and return to shore. Due to these behaviors’ partial successes, we expected optimal behaviors to include aspects of OVM and DVM, but with changes to promote nearshore retention and feeding throughout dispersal, if possible.

### Optimal Swimming Behaviors

We used dynamic programming to compute optimal swimming policies for larvae under five biological scenarios and eight environmental scenarios (Figs. 3 and S4.1$-$4.8). The biological scenarios included feeding and nonfeeding larvae with a 20-day larval duration; feeding larvae with the same larval duration, but spawned with insufficient energy for maintenance through dispersal; and feeding larvae with 6-day and 80-day larval durations. The environmental scenarios included all triplets of nearshore versus diurnal predation, still water versus upwelling, and low versus high food abundance (or energy surplus size, for nonfeeding larvae). See Table 1 for specific parameter values. These scenarios, although not exhaustive of the diversity in nature, covered a broad range of conditions, each with rich and unique outputs. To keep our analysis concise, we put visualizations for most scenarios in Supplement 4. Key similarities and differences across scenarios are summarized below and in Table 2.

A striking similarity across optimal trajectories in most scenarios was the tendency to visit the surface toward the beginning of dispersal, rather than the end (Figs. 3, row I). This trend emerged for multiple reasons, such as using surface currents to escape nearshore predation (Fig. 3A–B.II), avoiding surface currents that may transport larvae away from the nearshore habitat before settling (Fig. 3B.II and D.II), accumulating sufficient energy for metamorphosis (Fig. 3, row III), and, when larvae are spawned with insufficient energy for maintenance, gathering food to avoid starvation (Figures S4.1–8, rows C and E). Surface visits later in dispersal were more sporadic, and were generally for either staying offshore of the dangerous nearshore habitat (Fig. 3B.II) or replenishing energy before settling (Fig. 3A.III). The optimality of these front-loaded behaviors provides a theoretical justification for the commonness of the OVM and Hybrid archetypes in nature, and particularly in upwelling regimes.

**Table 2:** Summary of how optimal vertical swimming behaviors differed across several environmental and biological variables

Predation	Current	Notes on Optimal Larval Trajectories
Nearshore	Still Water	Overall Description: Optimal trajectories usually visited surface at start of development to diffuse away from nearshore predators, and returned occasionally for feeding or to diffuse toward shore (especially near end of development)
		Similar Archetypes: None
		Figures: 3A, S3.1, S3.2
		Effects of Nutritional Mode: No visible effects
		Effects of Energy Limitations: With more abundant food or more energy at spawning, optimal trajectories perform shorter, more frequent surface visits
		Effects of Larval Duration: Optimal trajectories with 6-day larval durations may spend entire duration in surface to acquire energy for metamorphosis. Those with 80-day durations visit the surface often throughout dispersal to avoid starvation
Nearshore	Upwelling	Overall Description: Optimal trajectories visit surface at start to advect away from predators, and occasionally during dispersal to feed or maintain position away from shore
		Similar Archetypes: OVM
		Figures: 3B, S3.3, S3.4
		Effects of Nutritional Mode: Optimal trajectories for nonfeeding larvae stopped visiting the surface at end of development to avoid offshore transport. Feeding larvae with energy limitations visited surface until end of development, despite offshore transport
		Effects of Energy Limitations: Greater energy availability (food or amount at spawning) promoted shorter, more frequent surface visits. See also Nutritional Mode
		Effects of Larval Duration: Optimal trajectories with 6- and 80-day durations were more likely to visit surface at end of development than those with 20-day durations
Diurnal	Still Water	Overall Description: Surface visits almost exclusively at night, and more often at the start of dispersal
		Similar Archetypes: DVM, Hybrid
		Figures: 3C, S3.5, S3.6
		Effects of Nutritional Mode: Optimal trajectories of nonfeeding larvae visited surface at end of dispersal, rather than start, to achieve onshore diffusion
		Effects of Energy Limitations: Trajectories of feeding larvae visited the surface more often when spawned with less energy or when food was limited. This combining these constraints resulted in an optimum similar to strict DVM
		Effects of Larval Duration: Optimal trajectories with 6-day durations remained in the surface day and night when food was scarce
Diurnal	Upwelling	Overall Description: For feeding larvae, optimal trajectories visited the surface nocturnally to gain energy at the start of dispersal, and performed additional nocturnal visits as needed to avoid starvation or prepare for metamorphosis
		Closest Archetypes: DVM, Hybrid
		Figures: 3D, S3.7, S3.8
		Effects of Nutritional Mode: Nonfeeding larvae had no incentive to visit the surface at all
		Effects of Energy Limitations: Optimal trajectories of larvae with limited food and spawned with insufficient energy visited the surface throughout dispersal in a DVM-like fashion, despite significant offshore transport. Otherwise, trajectories ceased regular surface visits a few days into dispersal
		Effects of Larval Duration: Over a 6-day larval duration, optimal trajectories struggled to quickly gather energy for metamorphosis while avoiding offshore transport and diurnal predation

See Table 1 for the parameters used in each scenario. For more details and visualizations of the optimal larval behaviors described here, we refer the interested reader to Supplement 3

Predation scheme had the most noticeable effect on optimal larval swimming behaviors. Diurnal predation typically resulted in optimal larval trajectories that visited the surface almost exclusively at night, resembling the DVM and Hybrid archetypes (Fig. 3C–D.I). In contrast, optimal trajectories subject to nearshore predation freely visited the surface at any time, often staying for multiple days (Fig. 3A–B.I). Under upwelling conditions, these differences in vertical movement resulted in greater offshore movement given nearshore predation (Figs. 3B.II and D.II). In both current regimes, nearshore predation also resulted in larval trajectories that were more likely to end dispersal with adequate energy, $E_T > 1$, due to uninterrupted feeding opportunities and fewer vertical migrations (Fig. 3, row III). In fact, the need to gather food quickly drove optimal trajectories for short-lived larvae with limited food to reside in the surface continually despite diurnal predation.

Optimal behaviors were often similar across current regimes (Figs. 3, row I), but their resulting trajectories differed dramatically with respect to cross-shore transport (Figs. 3, row II). The directed currents in an upwelling regime allowed optimal larval trajectories to deliberately move toward or away from shore at specific moments, which was beneficial for avoiding nearshore predation (Figs. 3B.II and S4.3–4). However, this current was generally disadvantageous given diurnal predation, with offshore transport occurring as a byproduct of feeding in the surface (Figs. 3D.II and S4.7–8). As intuition might suggest, optimal trajectories for nonfeeding larvae in upwelling and diurnal predation did not visit the surface at all (Figs. 3S4.7–8, row B).

On the other hand, larvae in still water had no mechanism for reliably achieving cross-shore transport, and instead used the greater diffusivity of the surface layer to increase their chances of moving in the correct direction. In still water with nearshore predation, for instance, optimal trajectories of both feeding and nonfeeding larvae visited the surface at the start of dispersal (Figs. 3A.I and S4.1–2, rows A–B). This similarity across nutritional modes suggested that this surface visit was not solely for feeding, but also for cross-shore transport. Additionally, nonfeeding larvae in still water with diurnal predation was the only scenario we considered where optimal trajectories only visited the surface at the end of dispersal (Figures S4.5–6, row B). These nocturnal surface visits were gambits for achieving fast onshore diffusion, compensating for slow offshore diffusion throughout development.

Optimized vertical swimming behaviors bore many similarities to the three active swimming archetypes in Fig. 2. Behaviors similar to OVM (Fig. 2B.I), or at least with surface visits concentrated at the start of dispersal, were optimal in many scenarios with nearshore predation (Figs. 3A–B.I and S4.1–4). Behaviors similar to the Hybrid archetype were common among optimal larval trajectories subject to diurnal predation (Figs. 3C–D.I and S4.5–8). Behaviors similar to strict DVM were uncommon, only emerging as optimal controls for feeding larvae spawned with insufficient energy for maintenance and subject to limited food and diurnal predation (Figures S4.5 and S4.7, rows C and E). In these cases, even optimal trajectories rarely finished dispersal close to shore, so strict DVM was still far from advantageous.

**Fig. 4:**
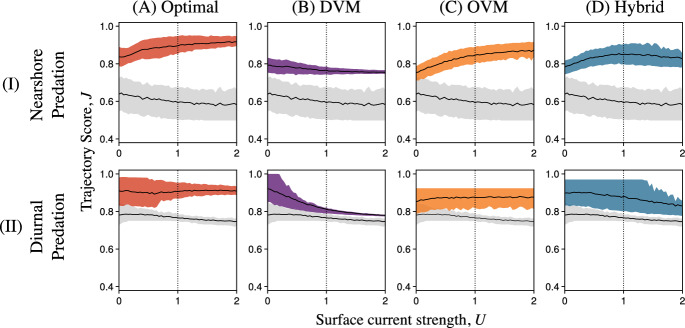
Relationships between Trajectory Scores, *J*, and upwelling current velocities, *U*, for **A** optimal vertical swimming behaviors (red), **B** the DVM archetype (purple), **C** the OVM archetype (orange), **D** the Hybrid archetype (blue), and passive drifting (gray in all panels). We considered both the nearshore and diurnal predation schemes (Rows I and II, respectively). Parameters besides *U* were held at the default values in Table 1. At $U = 0$ ($U = 1$, vertical dotted lines), conditions were identical to those in Figs. 3A and C (B and D) (Color figure online)

**Fig. 5:**
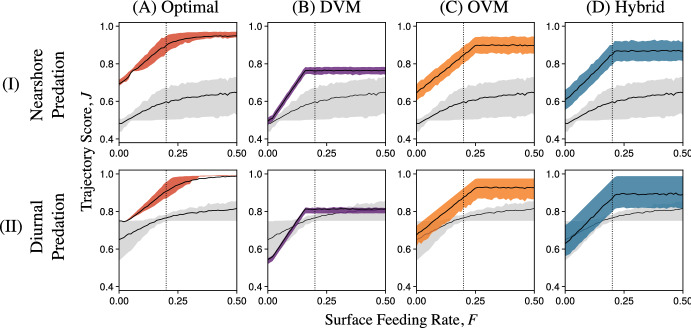
Relationships between Trajectory Scores, *J*, and feeding rate (a proxy for food abundance), *F*, for **A** optimal vertical swimming behaviors (red), **B** the DVM archetype (purple), **C** the OVM archetype (orange), **D** the Hybrid archetype (blue), and passive drifting (gray in all panels). We considered both the nearshore and diurnal predation schemes (Rows I and II, respectively). Parameters besides *F* were held at the default values in Table 1. At $F = 0.2$ (vertical dotted lines), conditions were identical to those in Fig. 3B and D (Color figure online)

### Comparing Optimal and Archetypal Behaviors

#### Environmental Variability

In nature, even larvae of the same species are likely to encounter different currents and food or predator abundances across time and space. The optimal behaviors described in the previous section were finely tuned to specific conditions and require larvae to access an unrealistic amount of information about their surroundings. However, vertical swimming behaviors like the OVM, DVM, and Hybrid archetypes may be common not because they are optimal, but because they allow adequate survival through metamorphosis under many conditions and only require information that is readily available to larvae (e.g., light–dark cues). We explored this hypothesis by using the Trajectory Score *J* to quantify how well these archetypes promoted survival over different upwelling strengths, *U*, feeding rates, *F* (a proxy for food abundance), and predator distributions (nearshore or diurnal). As in Section 3.1, we focused on the case of feeding larvae with the default parameters in Table 1. However, we repeated this analysis for nonfeeding larvae and feeding larvae spawned with insufficient energy in Supplement 4.2. Important differences are reported below.

Reassuringly, the behavioral archetypes we considered produced larval trajectories with greater scores than passive drifting in nearly all scenarios, showing the potential benefits of vertical swimming in numerous circumstances (Figs. 4, 5 and S4.1-2). Furthermore, we obtained the greatest mean Trajectory Scores in each current regime, food abundance, and predation scheme using the optimal policy computed through dynamic programming. The one exception to this rule occurred for larvae with diurnal predation, weak upwelling, and sufficient energy for maintenance at spawning— in this scenario, the DVM archetype performed slightly better than the computed optimum (Fig. 4B.II; more visible in Figure S4.1). We suspect that dynamic programming found a nearly optimal behavior in this case (see Fig. 3C.I), but did not find the DVM archetype we proposed due to numerical errors related to discretization and interpolation.

Nonetheless, the DVM archetype was clearly well suited to these conditions. On the other hand, DVM resulted in lower Trajectory Scores than the OVM and Hybrid archetypes with stronger currents, regardless of the larva’s starting energy and nutritional mode (Figs. 4B–D and S4.3–4). In these cases, visits to the surface late in dispersal resulted in offshore advection that carried trajectories away from the nearshore post-larval habitat (e.g., Fig. 2C.II). The fitness gained through diel vertical migrations appeared quite sensitive to the strength of the upwelling current (see Supplement 4.3 for more DVM-like examples).

Unlike the DVM archetype, the OVM and Hybrid archetypes produced larval trajectories with scores that were insensitive to the upwelling current’s strength under the diurnal predation conditions (Figs. 4C-D.II). This was because we parameterized these two behaviors to result in equal offshore and onshore displacement, regardless of current strength strength. With nearshore predation, this choice resulted in the opposite trends between *U* and Trajectory Scores: DVM performed similarly well regardless of current strength, while OVM performed better with stronger currents that helped larvae escape nearshore predation (Figs. 2B and 4B–C.I). The relationship between the Hybrid archetype’s Trajectory Scores and current strength increased over lower values of *U* for the same reason (Fig. 4D.I). For greater values of *U*, the Hybrid archetype may have returned larvae to the dangerous nearshore habitat too early, slightly reducing fitness. Still, the Hybrid archetype was nearly optimal in weak to moderately strong upwelling, while the OVM archetype was nearly optimal for moderate to strong upwelling (Figs. 4C–D and S4.1).

Unlike current strength, food abundance *F* had a positive effect on Trajectory Scores for all behaviors of feeding larvae we considered, including optimal and Passive ones (Figs. 5 and S4.2). For larvae spawned with sufficient energy for maintenance, Trajectory Scores for the DVM, OVM, and Hybrid archetypes increased linearly over low values of *F*, eventually reaching thresholds past which further increases in food abundance did not affect Trajectory Scores (Figs. 5B–D.I). This threshold represented the food abundance at which larvae can reliably finish dispersal with at least 1 unit of energy. This threshold was least for DVM ($F \approx 0.15$, Fig. 5B), where larvae could feed until the end of dispersal but frequently failed to arrive in the nearshore (Fig. 2B.II). This threshold was higher for the Hybrid archetype ($F \approx 0.2)$, in which larvae fed until day $t = 12$, and highest for the OVM archetype ($F \approx 0.25$), in which larvae fed until day $t = 8$ (Figs. 2C–D and 5C–D). Expected Trajectory Scores above these thresholds had the same ordering (Figs. 5B–D). This implied that in nature, variability in food abundance above $F = 0.25$ would favor OVM, while variability above $F = 0.15$ could favor any of the three archetypes we considered. The best vertical swimming behavior may be determined by the worst possible conditions.

#### Organismal Priorities

Although our above analyses treated avoiding predation and starvation, settling close to shore, and reserving energy for metamorphosis as equal priorities for survival (i.e., each weight $p_i = 0.25$), some of these requirements may be more or less important to different species. For instance, energy content may be more important than settling site for habitat generalists with stringent energy needs for metamorphosis, resulting in $p_\textrm{meta} > p_\textrm{settle}$. We found that changing these weights had only subtle effects on optimal larval trajectories (Supplement 5). However, it was instructive to visualize how well each behavioral archetype met these requirements for metamorphosis, especially when compared with optimal behaviors and passive drifting. The trajectory sub-scores, $J_\textrm{pred}$, $J_\textrm{starve}$, $J_\textrm{settle}$, and $J_\textrm{meta}$, provided a convenient framework for doing this. Again, we focused on feeding larvae with a 20-day larval duration spawned with sufficient energy for maintenance and dispersing in an upwelling current (defaults in Table 1). Starvation was nearly impossible under these conditions, resulting in $J_\textrm{starve} = 1$, so we limited this analysis to $J_\textrm{pred}$, $J_\textrm{settle}$, and $J_\textrm{meta}$. Results for nonfeeding larvae and feeding larvae spawned with insufficient energy (which sometimes starved and had $J_\textrm{starve} < 1$) are presented in Supplement 4, and important differences are highlighted below.

**Fig. 6:**
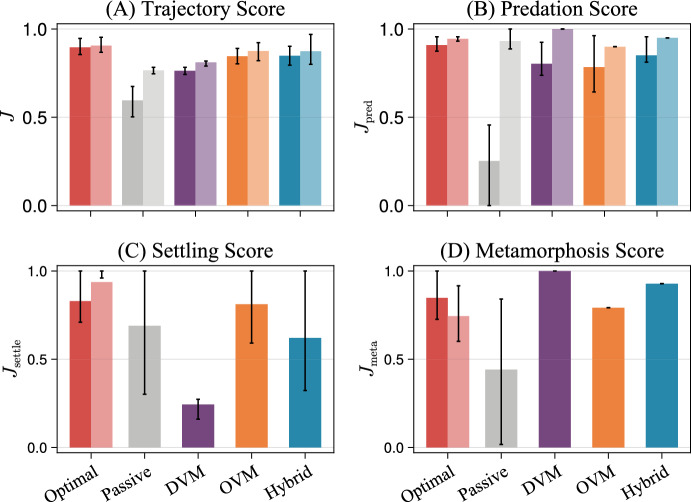
Success of the optimal swimming policy, passive drifting, and the DVM, OVM, and Hybrid archetypes with respect to **A** survival through metamorphosis, **B** avoiding predation, **C** settling close to shore, and **D** settling with enough energy for metamorphosis, all for larvae with the default parameters in Table 1. Bars represent mean scores, with dark (light) bars corresponding with the nearshore (diurnal) predation schemes. Settling and Metamorphosis Scores did not depend on predation schemes, except that optimal behaviors were different for each scheme. Error bars represent interquartile ranges— note that in (**C**), the mean Settling Score for the optimal behavior with diurnal predation was below the first quartile (color figure online)

In both the nearshore and diurnal predation schemes, expected total Trajectory Scores *J* were greatest for the optimal behavior and lowest for passive drifting (Fig. 6A). The three archetypes scored in the middle, with similar Trajectory Scores produced by OVM and Hybrid in each predation scheme and lower scores produced by DVM. The difference between passive drifting and the three archetypes was small in diurnal predation but large in nearshore predation, since passively drifting larvae rarely visited the surface and therefore experience limited opportunities for offshore transport (Fig. 2A).

However, different behavioral archetypes did better with respect to different larval requirements, resulting in different orderings for the three subscores $J_\textrm{pred}$, $J_\textrm{settle}$, and $J_\textrm{meta}$. For instance, given diurnal predation, trajectories produced using DVM were better at avoiding predation (i.e., received greater scores $J_\textrm{pred}$) than those produced using OVM, Hybrid, and even the optimal behavior. Given nearshore predation, DVM and OVM performed surprisingly similarly with respect to predator avoidance, while Hybrid performed slightly better on average (Fig. 6B).

The OVM archetype produced larval trajectories that more frequently delivered larvae to the nearshore habitat (i.e., received greater scores $J_\textrm{settle}$) than other non-optimal behaviors (Fig. 6C). The Hybrid archetype performed similarly well to passive drifting in this respect, while the DVM archetype was much less effective. This was not surprising, given the offshore distance trajectories $X_t$ shown in Fig. 2B.II. On the other hand, larval trajectories differed substantially within the OVM, Hybrid, and Passive behaviors, as evidenced by the large error bars on $J_\textrm{settle}$ in Fig. 6C. Biologically, this suggested that even in the same environment, larvae performing identical swimming behaviors may still follow trajectories leading to very different fates (e.g., successful settling versus offshore loss).

Finally, regardless of predation risk and final settling position, the DVM archetype was the most successful behavior at reserving energy for metamorphosis (i.e., received the greatest scores $J_\textrm{meta}$), with DVM-driven trajectories ending dispersal with more energy than even optimal ones, on average (Fig. 6D). Hybrid was slightly better than OVM in this regard, since it allowed larvae to feeding closer to the end of dispersal. Energy subscores $J_\textrm{meta}$ did not vary across trajectories within the DVM, OVM, and Hybrid archetypes because food access was completely determined by time spent in the surface, which was prescribed. In contrast, energy subscores varied somewhat across optimized larval trajectories, which responded to larvae’s stochastic states, and varied greatly across passively drifting ones that had no mechanisms of depth control (Fig. 6).

Overall, the OVM behavioral archetype appeared to be the most general of those considered. That is, the OVM behavior produced larval trajectories that similarly balanced predator avoidance (regardless of predation scheme), settling close to shore, and reserving energy for metamorphosis. The Hybrid archetype was similarly general, but slightly better at ensuring that larvae settled with sufficient energy and slightly worse at returning larvae to shore. The DVM archetype was more specialized, resulting in excellent predator avoidance and energy gathering but exporting larvae farther offshore. As shown in Supplement 4.3, however, different implementations of DVM resulted in different specializations. For instance, spending half as much time in the surface each night resulted in larval trajectories that consistently settled close to shore, but with less energy reserved for metamorphosis.

## Discussion

We used a simple mathematical model of marine invertebrate larval development to illustrate that weak locomotion can improve fitness during dispersal if it allows propagules to benefit from environmental heterogeneity. We measured the fitness of larval behaviors using a Trajectory Score that represented the probability of a larva following a given trajectory would survive through metamorphosis. The Trajectory Score quantified how well simulated larval trajectories balanced the conflicting needs of avoiding predators, avoiding starvation, and settling close to shore with sufficient energy for metamorphosis. Optimizing this score under a variety of conditions produced larval swimming patterns with several features in common with observed behaviors, such as ontogenetic and diel vertical migrations (OVM and DVM, respectively). These similarities suggested that our model successfully captured some of the trade-offs that have shaped larval swimming behaviors through natural selection.

Our model allowed us to compare the performance of three behavioral archetypes commonly reported in the literature— ontogenetic vertical migrations, diel vertical migrations, and a combination thereof (Hybrid)— against optimal and completely passive vertical movement. Different combinations of predator abundance, current strength, and food abundance favored different behaviors. For instance, diel vertical migrations were beneficial in weak currents and when predation occurred mainly in the surface during daylight, while ontogenetic vertical migrations and the Hybrid behavior were better in strong upwelling and when predation mainly occurred near the shore. Each behavior achieved a different balance of predation risk, nearshore settling, and energy conservation, but no behavior (besides the optimum) outperformed the others in all three categories. Together, our results indicated that the most suitable behavior for larvae of a given species depends on both environmental factors and larval biology and ecology.

This modeling experiment was a case study on how propagules with weak locomotive abilities can dramatically alter their fates by exploiting the structure of their environment, as well as the trade-offs they face in doing so. For coastal marine larvae, the directed currents associated with upwelling circulation provide opportunities to reliably achieve on- and offshore transport at convenient moments during dispersal. Larvae in still water— in which cross-shore movement is diffusion-driven, and therefore random and bidirectional— enjoy no such opportunities. However, directed currents are a double-edged sword. Larvae exhibiting behaviors not finely tuned to their environment risk being washed offshore (Meyer et al 2021). The literature contains several accounts of larvae exhibiting different behaviors throughout dispersal (or between populations) to better navigate their surroundings. For instance, the megalopae (mature larvae) of the estuarine crab *Carcinus maenas* achieve transport into into estuaries through flood-phased tidal vertical migrations (Queiroga et al 2007; Zeng and Naylor 1996b). Younger larvae of the same species achieve offshore transport through ebb-phased tidal vertical migrations in the bays of North Wales, United Kingdom (Zeng and Naylor 1996a), but diel vertical migrations off the west coast of Portugal (Queiroga et al 2007). This example illustrates how propagules’ adaptations and behavioral plasticity in response to environmental features improve fitness and allow populations to expand geographically.

Larvae and other propagules contend with environments that change over time predictably (e.g., daily and seasonally) and unpredictably (e.g., variability and extreme events). Adults can limit the exposure of their offspring to predictable variability by timing their reproduction with favorable conditions (Donahue et al 2015; Morgan 1995), and larvae can, to some extent, cope with unpredictable variations through behavioral and developmental plasticity (Miller and Morgan 2013; Boidron-Metairon 1988; Strathmann et al 1993). Additionally, we illustrated that some larval behaviors widely reported in the literature performed well, if not optimally, in a variety of environments. For example, the ontogenetic vertical migration and Hybrid behaviors performed equally well in still water and strong upwelling when predation was assumed to occur mainly in the surface during daylight. Similarly, the performance of the diel vertical migrations behavior was insensitive to changes in food abundance. It is clearly advantageous for propagules to be capable of successful dispersal despite environmental fluctuations. In fact, it is likely that natural selection has favored behaviors that maximize success in the worst, most extreme conditions, rather than in typical ones (Donahue et al 2015). Comparing larval swimming patterns that enhance population persistence over several generations to those presented here would be an interesting direction for future research.

The best behavior for a larva depends on both environmental factors and the ecology and biology of its species. Consequently, diverse behaviors have been documented among larvae of different species within in the same coastal environment. For instance, Bonicelli et al (2016) studied the vertical migrations of several species of barnacles and bivalves off the west coast of Chile, and noted that some species preferred different depths during diel vertical migrations, while others’ depth profiles appeared not to change over time at all. This diversity may be attributable, in part, to species-specific differences in predator–prey ecology, habitat specificity, and energetic requirements. Based on our results, species experiencing greater diurnal predation or costly metamorphoses may be more likely to perform diel vertical migrations, while those with a strong preference for settling close to shore would be less likely to do so in an upwelling regime. However, this behavioral diversity may be due to other factors not included in our analysis, such as larval morphology, development time, and perhaps competition between larvae (Shanks et al 2003; Morgan 1995; Young 1995).

Given the apparent ubiquity of diel vertical migration in nature, we were surprised by the narrow set of environmental conditions over which our archetype outperformed other behaviors. This result should be interpreted with the following caveats. First, we found that diel vertical migrations were clearly advantageous with respect to predator avoidance and gathering energy for metamorphosis. For species for which settling close to shore is a low priority compared with these other requirements (e.g., habitat generalists), diel vertical migrations could be an ideal behavior. Second, our main text only considered a single idealized version of this behavior, in which larvae spent six hours per night in the surface. Past studies by Meyer et al (2021), James et al (2019), and Sundelöf and Jonsson (2012) have shown that small changes in how diel vertical migration behaviors are modeled may result in large changes to apparent fitness. We showed in Supplement 3 that different formulations of this behavior sometimes resulted in better performance across the scenarios we considered. Finally, this analysis considered only a small range of coastal environments and documented larval behaviors. Upwelling circulation is common on eastern oceanic boundaries worldwide (Chavez and Messié 2009), but there exist several other ecologically important flow patterns (e.g., downwelling and tidal circulation) that could also favor diel vertical migrations and similar behaviors.

It is probable that our model did not capture some advantages of diel vertical migrations and other behaviors due to our coarse descriptions of the environment and larval biology. We assumed that predator and food abundance and current strength varied across distinct depth layers, rather than continuously throughout the water column. In reality, larvae may migrate to specific depths at different moments during dispersal, depending on their instantaneous and long-term goals (e.g., onshore transport versus feeding). In our model, larvae were only able to experience the average conditions in each layer. We also assumed that larvae developed for a fixed duration of *T* days, and that their predation risk and energetic needs did not change over time. The effect of size on predation over time differs across species, with individuals of some species experiencing increased predation due to greater visibility and individuals of other species experiencing reduced predation due to size refuge (Allen 2008; Rumrill 1990; Pechenik 1999). While our Trajectory Score weighed predation equally throughout dispersal, real larval behaviors may be shaped by differences in risk throughout development. Finally, while modeling behavioral archetypes, we assumed that all larvae executed those behaviors perfectly. However, data suggest that while populations of larvae tend to prefer different depths over time, individuals within populations do not behave identically (Bonicelli et al 2016; Queiroga et al 2002; dos Santos et al 2008; Shanks 1986; Tapia et al 2010). This variability may be adaptive in ways our model does not capture. For example, differences in how larvae with a brood swim may result in an individual’s offspring experiencing diverse conditions during dispersal and settling, increasing the probability that at least some will survive through metamorphosis (Meyer et al 2021). The assumptions of a two-layer environment, constant predation, and identical behaviors significantly simplified this broad analysis of vertical swimming behaviors. However, we suspect that relaxing these assumptions would result in interesting and nuanced refinements of our results.

Finally, it is important to acknowledge the limitations of our optimization approach. Dynamic programming is a powerful tool for understanding what behaviors may be favorable under certain assumptions and conditions, but it cannot predict what traits might actually emerge in a population due to mutation and selection (Mangel and Clark 1988). Furthermore, our focus on successful larval trajectories neglected possible multigenerational and population-scale benefits of planktonic development and larval swimming, including range expansion, gene flow, and metapopulation connectivity and resilience (Burgess et al 2016; Hedgecock 1986; Levin 2006; Pechenik 1999; Shaw et al 2019; Strathmann 1974), as well as risk-spreading benefits mentioned in the previous paragraph. Last, because we used the probability of survival through metamorphosis to combine the conflicting needs of larvae to avoid predation and starvation and settle under good conditions, optimization could have produced trajectories that maximized some of these goals at the cost of others. In practice, this only happened under extreme conditions in which simulated larvae could not gather adequate food without experiencing unacceptable offshore transport (e.g., Figures S3.3E and S3.7E). A rigorous reachability analysis would be useful for identifying these conditions *a priori* and generating hypotheses regarding combinations of environmental and larval traits (e.g., larval duration, energy content) that are incompatible in nature (Fleming and Rishel 1975).

These limitations notwithstanding, our results compellingly illustrate how propagules with limited locomotive abilities can exploit environmental features to great effect during dispersal. Regarding the biology of coastal marine larvae, our analysis suggests that commonly reported swimming behaviors promote successful metamorphosis under the right set of environmental and organismal conditions. More broadly, we argue that the assumption that propagules are passively moved by exogenous forces, as well as the methods by which active behaviors are modeled, must be carefully examined, especially when forecasting population dynamics.

### Supplementary Information

Below is the link to the electronic supplementary material.

Supplementary file 1 (pdf 4912 KB)

## Data Availability

Model code and simulated datasets generated during this study are available in the GitHub repository, https://github.com/alexdmeyer/optimal-larva-1.
