# Supplementary material for: Making Your Own Luck: Weak Vertical Swimming Improves Dispersal Success for Coastal Marine Larvae
Source: Bull Math Biol. 2024 Jan 28;86(3):23. doi: 10.1007/s11538-023-01252-2 (PMC10822797; doi:10.1007/s11538-023-01252-2)
Supplement: Supplementary file 1 — (pdf 4912 KB) [file 11538_2023_1252_MOESM1_ESM.pdf]

Making Your Own Luck: Weak Vertical Swimming Improves Dispersal Success for Coastal Marine Larvae
Supplementary Materials

Alexander D. Meyer, Alan Hastings, John L. Largier

November 9, 2023

Contents

1 Parameter Estimation 1
1.1 Distance-Based Quantities . . . 2
1.2 Energetic Quantities . . . 2
2 Passive Drifting 4
3 Optimal Larval Trajectories 6
3.1 Nearshore Predation Scheme . . . 6
3.1.1 Still Water with Nearshore Predation . . . 6
3.1.2 Upwelling with Nearshore Predation . . . 10
3.2 Diurnal Predation Scheme . . . 13
3.2.1 Still Water with Diurnal Predation . . . 13
3.2.2 Upwelling with Diurnal Predation . . . 16
4 Comparing Optimal and Archetypal Swimming Behaviors 19
4.1 Alternate Versions of Figures 4 and 5 . . . 19
4.2 Different Biological Scenarios . . . 20
4.2.1 Nonfeeding Larvae . . . 20
4.2.2 Spawning with Insufficient Energy for Maintenance . . . 23
4.3 Variations on Passive Drifting and DVM . . . 25
5 Weights of the Trajectory Score 30
6 Additional Simulations of Optimal Larvae 34

1 Parameter Estimation

Larvae of marine invertebrate species differ dramatically in their typical dispersal distances (Shanks, 2009; Shanks et al, 2003), energy budgets (Lucas et al, 1979; Sprung, 1984b; Thiyagarajan et al, 2003; Wendt, 2000), and habitat sizes (Nickols et al, 2015). By combining distance-based and energetic parameters into new, unitless parameters (that is, nondimensionalizing our model), we avoided tailoring our model to the biology of any

specific species. However, it was nonetheless important to choose physically and biologically justifiable values for these parameters. Those values were presented in Table 1 of the main text, and are justified below.

1.1 Distance-Based Quantities

All distance-based quantities, including current velocities and eddy diffusivity, were defined relative to the size of a typical adult coastal habitat, h, for the theoretical species of interest. Habitats sizes range from a few meters (e.g., for animals inhabiting small rocky reefs) to 10 km or more (e.g., for animals capable of surviving at various depths) (Nickols et al, 2015; Rasmuson, 2013). Shanks (1995) states that cross-shore current velocities, u, in coastal environments frequently fall in the range 0-25 km d^{-1}. Using the more conservative ranges $1 \leq h \leq 10$ km and $0 \leq u \leq 10$ km d^{-1} resulted in dimensionless velocities $U = u/h$ in the range 0-10 d^{-1}. This parameter had the greatest qualitative effect on optimal swimming behaviors in the range $0 \leq U \leq 2$, so we chose $U = 1$ as a default value for upwelling scenarios.

Largier (2003) and Nickols et al (2012) describe cross-shore eddy diffusivities, k, ranging from 10-100 km^2 d^{-1}. This resulted in dimensionless diffusivities $K = k/h^2$ in the range 0.1-100 d^{-1}. We chose the conservative default value $K = 0.2$ d^{-1} to obtain a system in diffusion did not dominate advection.

Finally, recall from the main text that in our two-layer upwelling system, the surface layer had offshore velocity $U_1 = U$ and cross-shore diffusivity $K_1 = K$, while the lower layer had velocity $U_0 = -\alpha U$ and diffusivity $K_0 = \alpha K$ for some constant α. The constraint $0 < \alpha < 1$ was intended to capture the typical upwelling scenario in which currents in the thin surface layer are stronger than those in the thick bottom layer, but the exact choice $\alpha = 0.25$ was arbitrary and did not qualitatively change our results.

1.2 Energetic Quantities

All energetic quantities, including the rates of energy uptake and expenditure on maintenance, growth, and locomotion, were defined relative to the energetic cost of metamorphosis, m. Establishing reasonable values for these quantities was particularly challenging because previously published studies differ in their study organisms, laboratory protocols, and reporting methods. We were unable to find complete energy budgets for any species; however, we based many of our parameter estimates on the studies of *Balanus balanoides* and *Mytilus edulis* published by Lucas et al (1979) and Sprung (1984a,b), respectively.

According to Lucas et al (1979), *B. balanoides* larvae require about $m = 125$ mJ of energy for metamorphosis, and may store up to an additional 210 mJ of energy for "swimming and exploration." This

suggests a maximum larval energy store of about $3m$ or more. We used $5m$ (that is, $E_{\text{max}} = 5$) to avoid overly constraining our optimization problem. In low food simulations, larvae rarely contained more than $3m$ mJ at once, while in high food simulations, larvae often reached their maximum of $5m$. However, the same results would be attained using a different choice of maximum and proportionally different definitions of "low" and "high" food abundance.

Lucas et al (1979) also observed that larva's 210 mJ energy surplus typically lasted 2.5 to 4 weeks, suggesting an average metabolic rate, g, of 7.5-12 mJ d^{-1}. We used the upper end of this range to obtain a dimensionless metabolic rate, $G = g/m$, of 0.1 d^{-1}. We chose dimensionless rates of energy uptake (that is, feeding), F, of similar magnitude to G so that modeled larvae would neither be forced to starve (if $F < G$) or easily meet their energetic needs (if $F \gg G$). The value $F = 0.2$ was a convenient default.

Finally, Sprung (1984b) suggested that *M. edulis* larvae may direct up to 20% of their energy expenditure toward locomotion while swimming at a rate of 2 mm s^{-1}. This suggests that the cost of swimming is $0.25G$ d^{-1}. The speed 2 mm s^{-1} is typical for larvae of many marine invertebrates (Chia et al, 1984). For the purposes of estimating the cost of a single vertical migration, we assumed that the water column had a uniform depth with a bottom layer 50 m thick and a surface layer 12.5 m thick. We assumed that, on average, vertical migrations transported larvae from the middle of one layer to the middle of the other, covering a distance of 31.25 m. A larva swimming or sinking at speed 2 mm s^{-1} would travel this distance in about 0.2 d, resulting in a total energy expenditure of

$$V = 0.2 \text{ d} \cdot 0.25G = 0.0045.$$

As a default, we rounded this value down to $V = 0.004$. However, we also assumed that modeled larvae were neutrally buoyant—rather than passively sinking from the surface to the bottom, they needed to actively swim downward. We assumed upward and downward vertical migrations both cost V units of energy. Since a modeled larva must perform a nearly equal number of upward and downward migrations, this is equivalent to modeling a larva for which swimming upward costs 0.008 units of energy, but swimming downward costs 0.

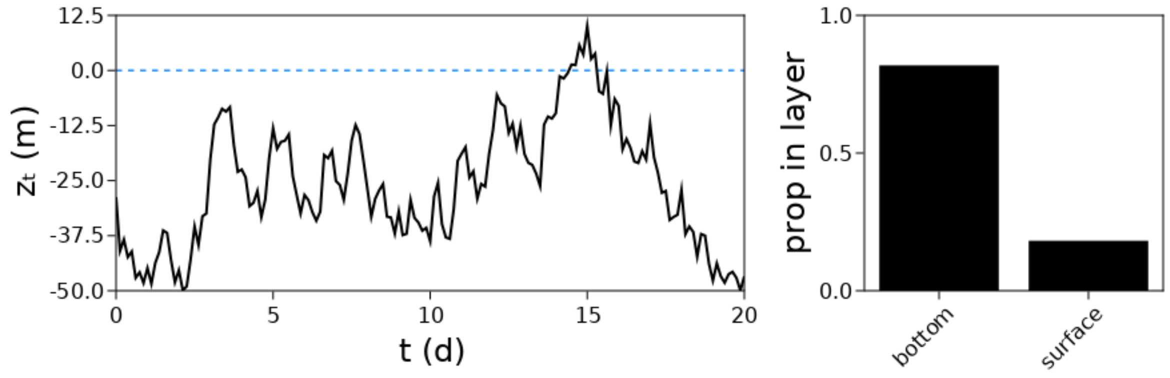

Figure S2.1: Left: A single realization of the depth, $z(t)$, of a passively floating larva. The blue dotted line, $z = 0$, separates the bottom and surface layers. Right: Proportions of the larval duration the process $z(t)$ spends in each layer, averaged over 1000 simulations.

2 Passive Drifting

In our two-layer upwelling scenario, the vertical position of a modeled larva takes one of two values: $Z_t = 0$ when the larva is in the bottom layer, and $Z_t = 1$ when the larva is in the surface layer. Passively drifting larvae were modeled as switching between these two states at random intervals (main text Figure 2A.I). These switches occurred according to an underlying random walk simulation in the water column. As in Supplement 1, suppose that the surface and bottom layers of the coastal environment have thicknesses 12.5 m and 50 m, respectively. Let $z(t) \in [-50, 12.5]$ represent the depth at time t of a larva in this environment, such that the underlying states $z(t) < 0$ and $z(t) > 0$ correspond with observed states $Z_t = 0$ and $Z_t = 1$, respectively. We assumed the larva was spawned from a random depth within the lower layer, and then changed depth over time according to a continuous-state random walk:

$$z(0) \sim \text{Uniform}([-50, 0]), \tag{2.1}$$

$$z(t + \Delta t) = z(t) + \sqrt{2k_z \Delta t}\zeta(t), \quad t = 0, \ldots, T - \Delta t \tag{2.2}$$

$$z = -50, 12.5 \text{ are reflecting boundaries.} \tag{2.3}$$

In equation (2.2), $\zeta(0), \ldots, \zeta(T - \Delta t)$ are independent standard normal random variables and k_z is the vertical eddy diffusivity of the coastal environment. In upwelling systems, the vertical eddy diffusivity is usually less than 100 m^2 d^{-1} (Barton et al, 2001; Hamilton and Jr., 1978; Haskell et al, 2015; Waldron and Probyn, 1991). We used this extreme value as the default, $k_z = 100$, because for smaller values of k_z, the process $z(t)$ rarely exited

the lower layer at all. Using $k_z = 100$, larvae spend about 18% of the larval duration in the surface. Because vertical advection is often very weak—Haskell et al (2015) reported upward velocities less than 1 m d^{-1} in the Eastern Tropical South Pacific upwelling system—we omitted it from our passive drifting model.

A single realization of this process and the expected proportion of the larval duration this process spends in each layer are shown in Figure S2.1.

3 Optimal Larval Trajectories

Results Section 3.2 in the main text discusses optimized larval trajectories in five biological scenarios and eight environmental scenarios. The biological scenarios included feeding and nonfeeding larvae with a 20-day larval duration; feeding larvae with the same larval duration, but spawned with insufficient energy for maintenance through dispersal; and feeding larvae with 6-day and 80-day larval durations. The environmental scenarios included all triplets of nearshore versus diurnal predation, still water versus upwelling, and low versus high food abundance (or energy surplus size, for nonfeeding larvae). Generalities and important similarities and differences across these scenarios are summarized in the main text and Table 2. However, Figure 3 in the text only shows results for one biological scenario (feeding larvae with 20-day larval duration) and four environmental scenarios (low food with either still water or upwelling and either nearshore or diurnal predation). Figures S3.1-S3.8 visualize optimized larval trajectories in all 40 combinations of biological and environmental scenarios, and the text in this supplement expands on the observations in Table 2.

3.1 Nearshore Predation Scheme

3.1.1 Still Water with Nearshore Predation

Optimal larval trajectories with these conditions are shown in Figures S3.1 (low food abundance) and S3.2 (high food abundance). Row A of Figure S3.1 is identical to main text Figure 3A.

General description and similar archetypes. Optimal trajectories always visit the surface at the start of dispersal. Surface visits occur throughout dispersal, and are particularly likely at the end of dispersal (unless the larval duration is short, $T = 6$ days). With no directed currents to contend with, optimal larval trajectories frequently finished dispersal within the nearshore habitat, $X_T < 1$. Since feeding larvae could visit the surface to feed without a large risk of offshore transport, no optimal trajectories hit starvation (that is, $E_t > 0$ for all $0 \leq t \leq T$) and all optimal trajectories finished dispersal with adequate energy for metamorphosis ($E_T \geq 1$). On the other hand, increased diffusion in the surface probably encouraged some movement away from nearshore predators and toward the nearshore habitat at the start and end of dispersal. On average, the optimal vertical swimming policy did not resemble any of the archetypes we considered.

Effects of nutritional mode. Optimal swimming policies were nearly identical for larvae spawned with adequate energy for maintenance (row A) and nonfeeding larvae spawned with adequate energy for maintenance

and metamorphosis (row B).

Effects of energy at spawning. When food was abundant, optimal trajectories for feeding larvae with 20-day larval durations were not strongly affected by energy at spawning (Figure S3.2, rows A and C). When food was limited, larvae spawned with less energy spent more time in the surface during the second half of dispersal (Figure S3.1C)

Effects of food abundance/surplus size. As noted in the previous point, the main effect of limited food in the surface was to encourage larvae to spend more time in the surface. With limited food, surface visits tended to be longer; in contrast, larvae with abundant food could gather sufficient energy through short, infrequent surface visits (compare columns I in Figures S3.1 and S3.2). For nonfeeding larvae, optimal trajectories were identical for larvae spawned with only enough energy for maintenance and metamorphosis compared with those spawned with an additional surplus (Figures S3.1B and S3.2B) probably due to the low estimated cost of vertical swimming derived in Supplement 1.

Effects of larval duration. For feeding larvae with a 6-day larval duration, optimal trajectories spent a large fraction of dispersal in the surface to quickly gather energy for metamorphosis—with low food abundance, trajectories may reside in the surface for the entirety of dispersal (Figures S3.1D and S3.2D). Optimal trajectories for feeding larvae with 80-day larval durations avoided starvation by spending a larger fraction of the second half of dispersal in the surface than larvae with a 20-day larval duration (Figures S3.1E and S3.2E).

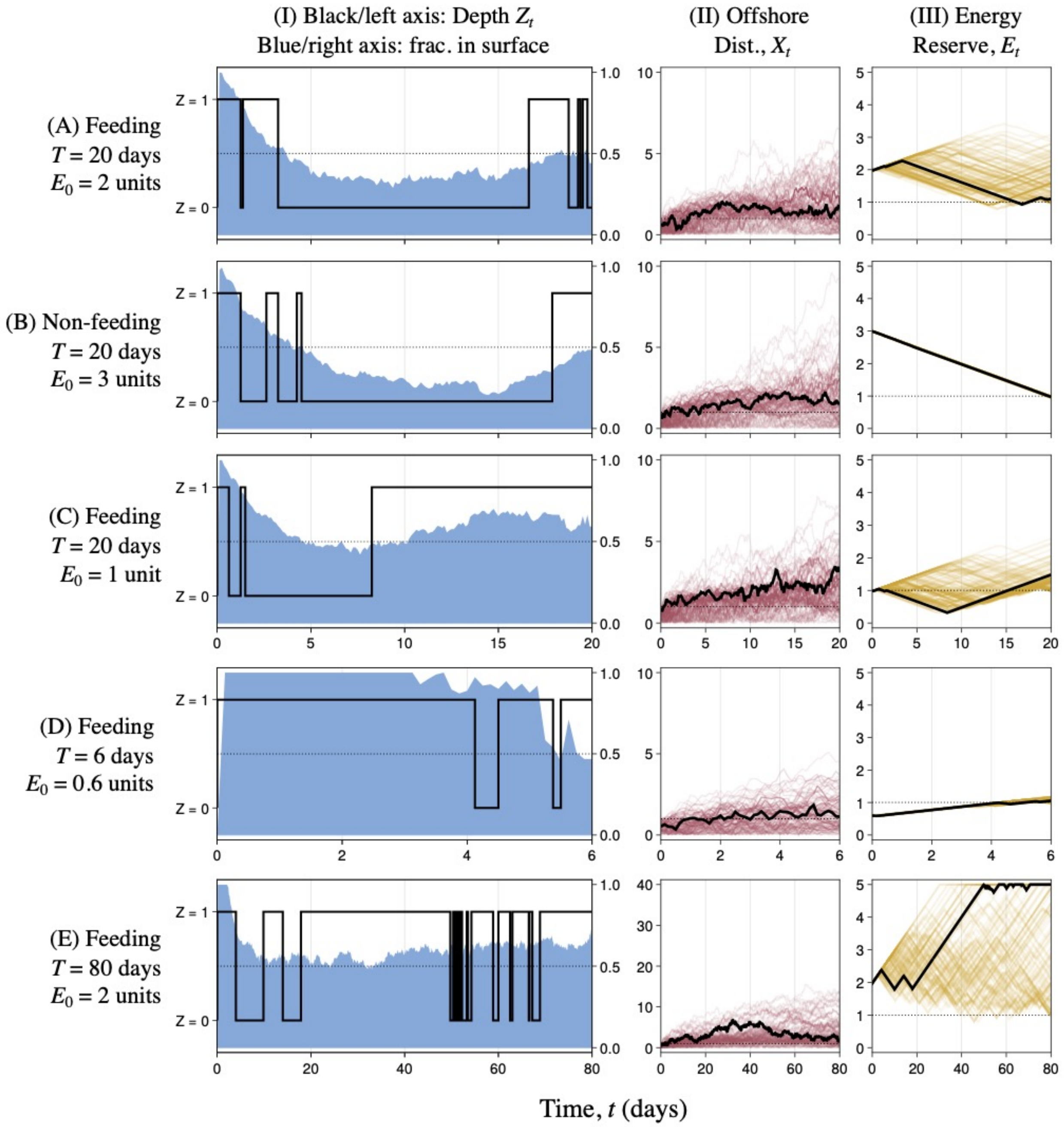

Figure S3.1: *Nearshore predation, still water, low food/small surplus.* Rows: (A) Default case of feeding larvae with 20-day larval duration spawned with enough energy for maintenance. (B) Nonfeeding larvae with 20-day larval duration and small surplus. (C) Feeding larvae with 20-day larval duration spawned with insufficient energy for maintenance. (D) Feeding larvae with 6-day larval duration spawned with enough energy for maintenance. (E) Feeding larvae with 80-day larval duration spawned with insufficient energy for maintenance. Columns: (I) Example depth trajectory Z_t (left axis) and proportion of larvae in the surface (right axis and blue shading). (II) Example offshore distance traectories, X_t. (III) Example energy trajectories, E_t. The black examples in II and III correspond with the same example simulation in I.

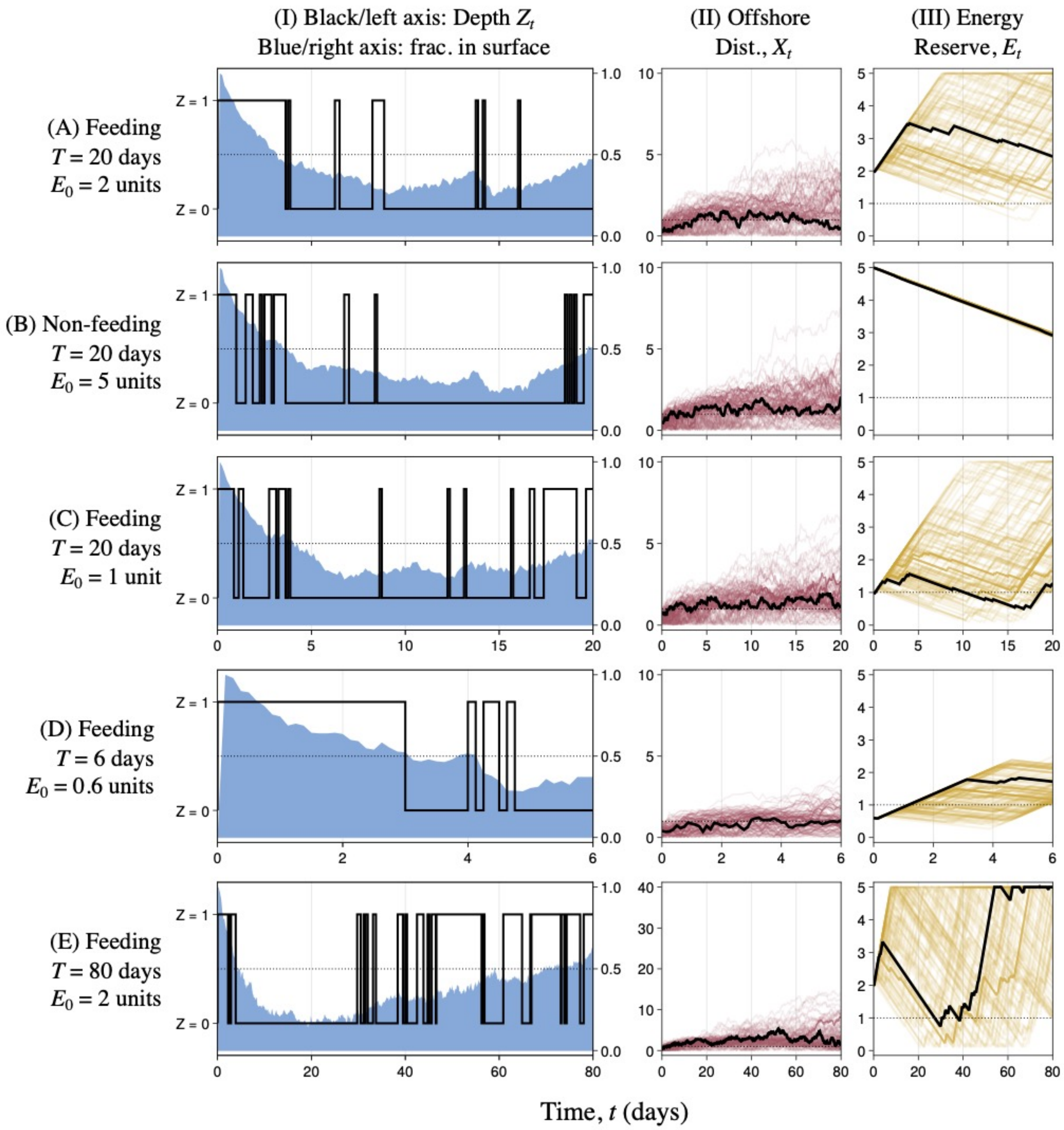

Figure S3.2: *Nearshore predation, still water, high food/large surplus.* Analogous to Figure S3.1, but row (B) shows nonfeeding larvae with a 20-day larval duration and a large energy surplus.

3.1.2 Upwelling with Nearshore Predation

Optimal larval trajectories with these conditions are shown in Figures S3.3 (low food abundance) and S3.4 (high food abundance). Row A of Figure S3.3 is identical to main text Figure 3B.

General description and similar archetypes. Optimal trajectories under these conditions resembled the OVM archetype in main text Figure 2B.I. Larvae visited the surface at the start of dispersal to achieve offshore transport away from nearshore predators. Throughout the remainder of dispersal, optimized larvae occasionally visited the surface to feed or maintain a safe offshore distance, but otherwise resided in the bottom layer to achieve transport toward the nearshore habitat.

Effects of nutritional mode. For both feeding and nonfeeding larvae with a 20-day larval duration, optimal dispersal trajectories began with a visit to the surface. This similarity indicated that the purpose of this visit was to achieve offshore transport, rather than to feed.

Effects of energy at spawning, food abundance, and surplus size. For nonfeeding larvae, a greater energy surplus at spawning favored fewer, shorter surface visits throughout dispersal aimed at maintaining a safe offshore distance. Regardless of surplus size, however, these visits ceased a couple days before settling to promote onshore transport (Figures S3.3B and S3.4B).

For feeding larvae, energy limitations (whether due to food abundance or energy at spawning) promoted surface visits until the very end of dispersal to avoid settling with insufficient energy for metamorphosis (Figures S3.3 and S3.4, rows A and D). When larvae were spawned without enough energy for maintenance throughout dispersal, optimal trajectories visited the surface at the end of dispersal even if it resulted in transport away from the nearshore habitat (Figures S3.3 and S3.4, rows C and E). It is not clear that a species whose larvae have these energy constraints could persist without abundant food in the environment.

Effects of larval duration. Over a larval duration of $T = 20$ days, optimal larval trajectories visited the surface for the first 2-10 days of dispersal on average, in an OVM-like fashion (Figures S3.3A-C and S3.4A-C). Over a shorter larval duration of only 6 days, larvae visited the surface at the start of dispersal for at least 2 days, and either remained there or returned later to feed, depending on food abundance (Figures S3.3D and S3.4D). Finally, over $T = 80$ days, trajectories consistently visited the surface for about 30 days in the lower food scenario and about 8 days in the higher food scenario (Figures S3.3E and S3.4E).

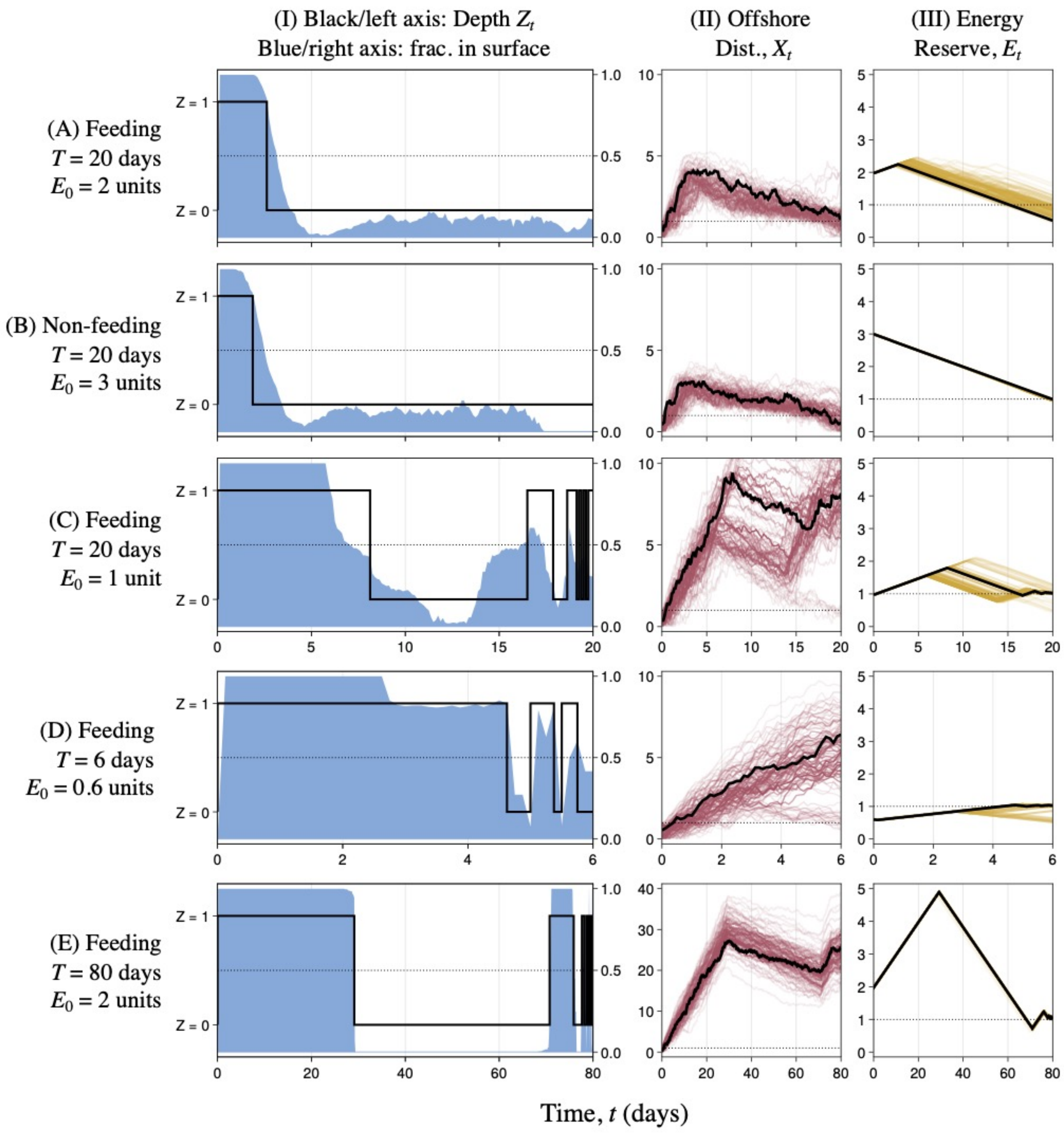

Figure S3.3: *Nearshore predation, upwelling, low food/small surplus.* Analogous to Figure S3.1.

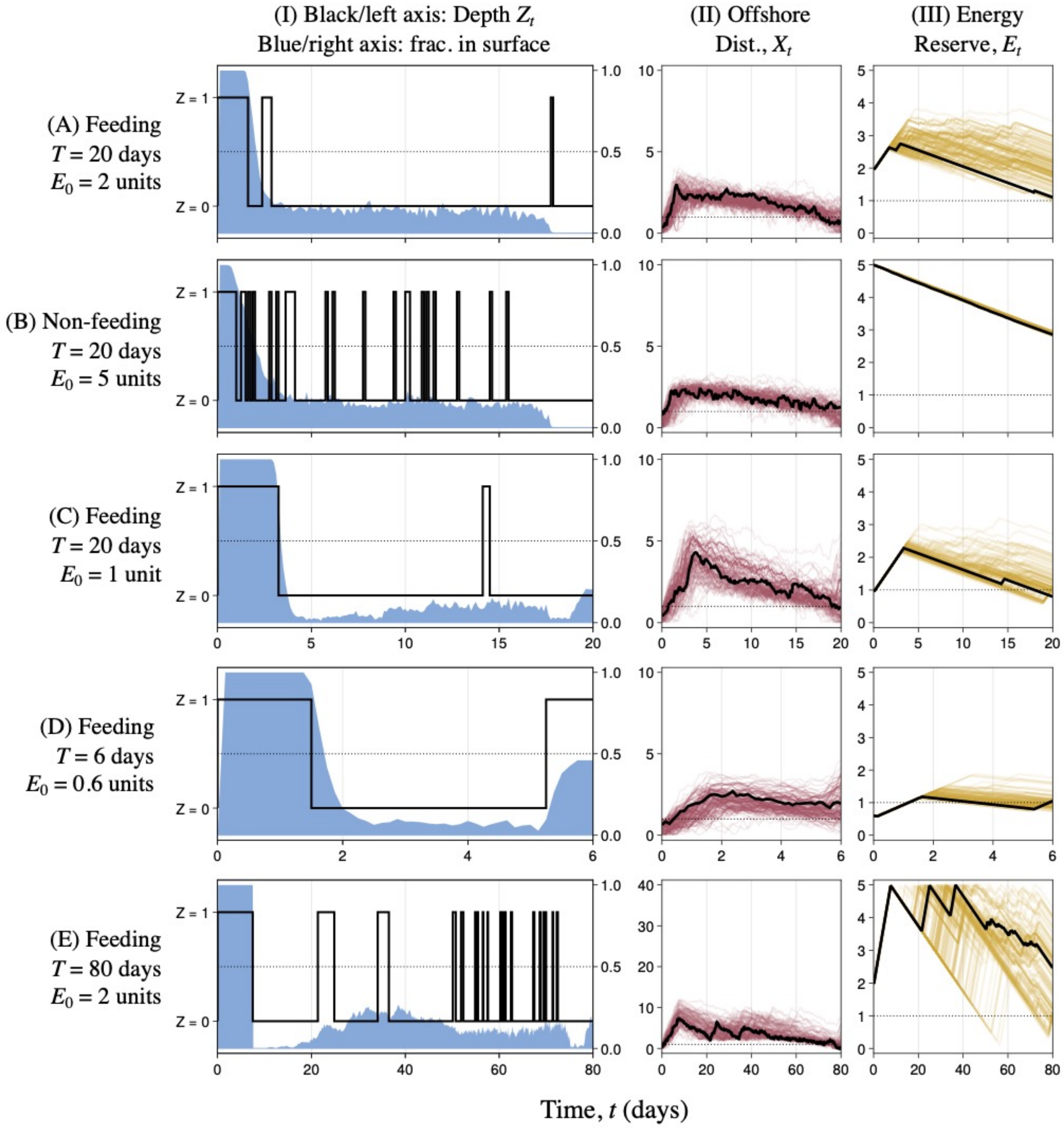

Figure S3.4: *Nearshore predation, upwelling, high food/large surplus.* Analogous to Figure S3.2.

3.2 Diurnal Predation Scheme

3.2.1 Still Water with Diurnal Predation

Optimal larval trajectories with these conditions are shown in Figures S3.5 (low food abundance) and S3.6 (high food abundance). Row A of Figure S3.5 is identical to main text Figure 3C.

General description and similar archetypes. Under these conditions, optimal larval trajectories almost exclusively visited the surface nocturnally. Corresponding larval swimming policies resembled, to varying degrees, either the DVM or Hybrid archetypes. In a few cases, optimal larval trajectories visited the surface nearly every night to feed. More often, however, these visits were concentrated near the start of dispersal.

Effects of nutritional mode. Nonfeeding larvae had little need to visit the surface. However, they tended to do so at the end of dispersal (rather than the beginning, as in the Hybrid behavior) to exploit the surface layer's greater diffusivity, increasing their chances of quickly drifting toward shore (Figures S3.5B and S3.6B). Optimal swimming policies for feeding larvae were very different due to the conflicting needs of predation and starvation avoidance and metamorphosis, resembling the DVM and Hybrid archetypes (Figures S3.5 and S3.6, rows A and C-E).

Effects of energy at spawning. Optimal trajectories of larvae spawned with insufficient energy for maintenance visited the surface nearly every night if food was scarce (Figures S3.5, rows C and E). This was necessary to avoid starvation and settling with insufficient energy. When food was abundant, optimal trajectories were more similar to the Hybrid behavior, regardless of energy at spawning (except over larval duration $T = 6$; see Figure S3.6, rows A, C, E).

Effects of food abundance/surplus size. Energy surplus size for nonfeeding larvae had a minimal effect on optimal trajectories (Figures S3.5B and S3.6B). For feeding larvae, more abundant food allowed optimal trajectories to cease visiting the surface nightly earlier into dispersal (Figures S3.5 and S3.6, rows A and C-E).

Effects of larval duration. Over the short larval duration $T = 6$ and with low food abundance, optimal trajectories remained in the surface for most of dispersal to gather enough food for metamorphosis (Figure S3.5D). Increased food abundance allowed larvae to gather energy more quickly, permitting a more DVM-like behavior (Figure S3.6D).

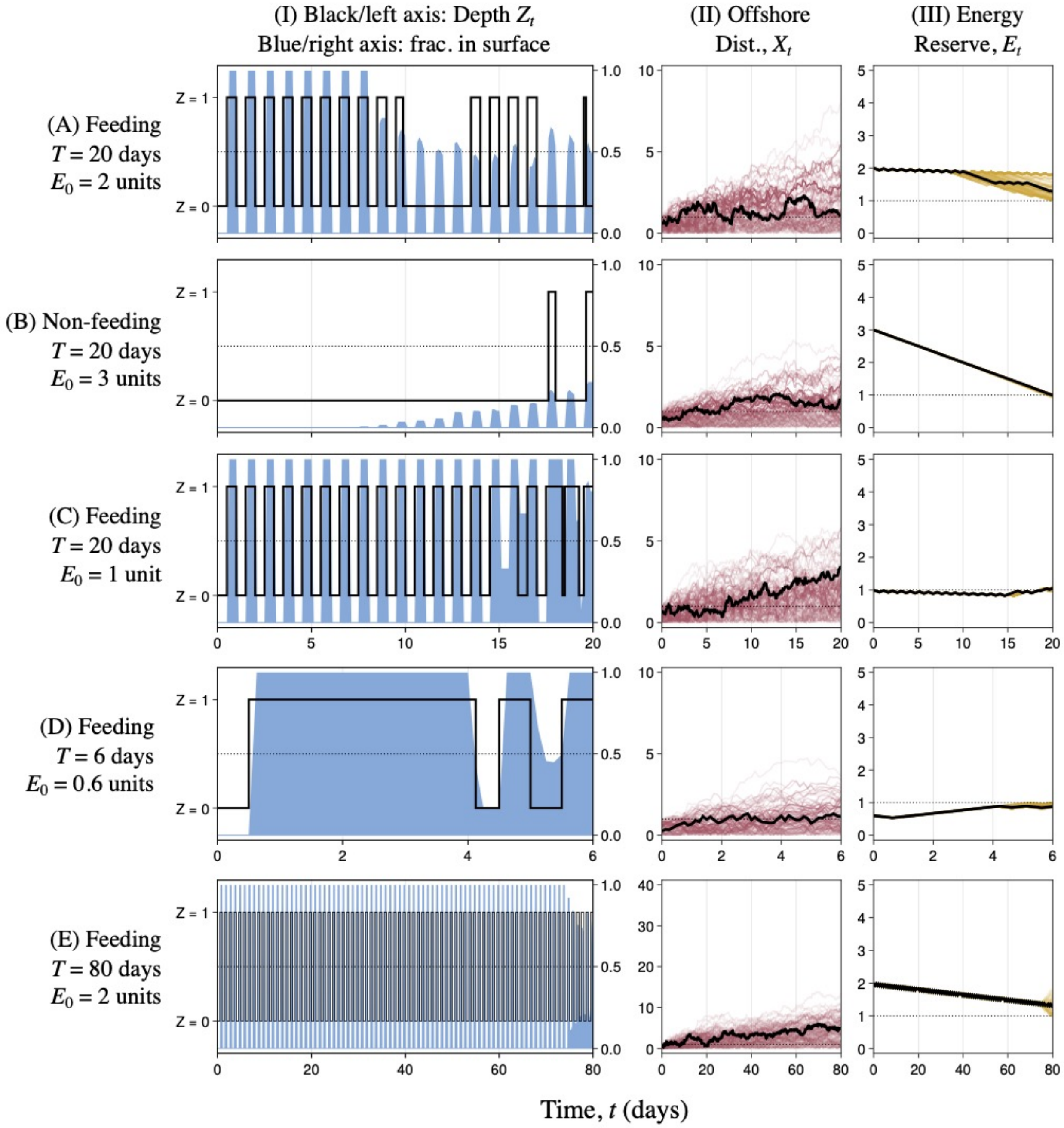

Figure S3.5: *Diurnal predation, still water, low food/small surplus.* Analogous to Figure S3.1.

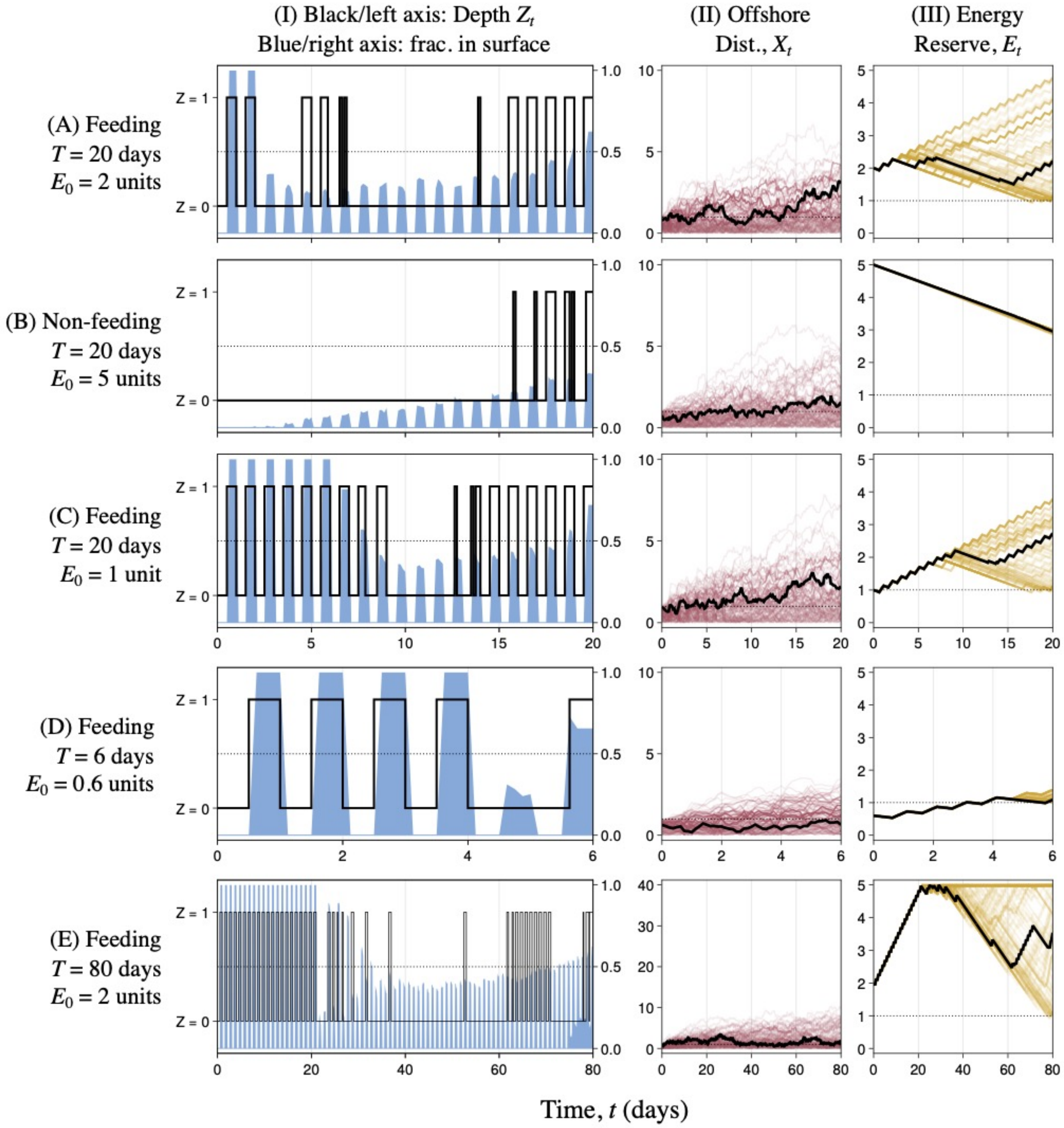

Figure S3.6: *Diurnal predation, still water, high food/large surplus.* Analogous to Figure S3.2.

3.2.2 Upwelling with Diurnal Predation

Optimal larval trajectories with these conditions are shown in Figures S3.7 (low food abundance) and S3.8 (high food abundance). Row A of Figure S3.7 is identical to main text Figure 3D.

General description and similar archetypes. Optimal vertical swimming policies under these conditions resembled the Hybrid and DVM archetypes, depending on energy availability (e.g., through food or surpluses at spawning). Larvae visited the surface nearly exclusively at night, and did so most often at the start of dispersal. Later surface visits allowed larvae to avoid starvation and settle with adequate energy for metamorphosis, but sometimes came at the price of settling far from shore (e.g., Figure S3.7, rows C and E).

Effects of nutritional mode. Nonfeeding larvae had no incentive to visit the surface under upwelling conditions: they received no benefit through feeding, and any possibility of diffusing toward shore was overpowered by the certainty of offshore advection (Figures S3.7 and S3.8, row B). Feeding larvae, on the other hand, were forced to visit the surface to gather energy (Figures S3.7 and S3.8, rows A and C-E), particularly when spawned with insufficient energy for maintenance during dispersal (rows C and E in the same figures).

Effects of energy at spawning. As in still water, insufficient energy at spawning resulted in a DVM-like optimal swimming policy unless food was abundant in the surface (Figures S3.7 and S3.8, rows C and E). When spawned with sufficient energy for maintenance throughout dispersal, optimal trajectories for larvae with a 20-day larval duration ceased regularly visiting the surface earlier (Figures S3.7 and S3.8, rows A and C).

Effects of food abundance/surplus size. Like energy at spawning, food in the surface allowed larvae to stop visiting the surface nightly earlier in dispersal (compare Figures S3.7 and S3.8). This was especially noticeable when larvae were spawned with insufficient energy for maintenance, as noted above.

Effects of larval duration. Most observations above are independent of larval duration. For larvae with a 20-day larval duration, optimal trajectories visited the surface at least the first 2-5 nights of dispersal, and continued to do so as needed to gather energy before settling (Figures S3.7 and S3.8, rows A and C). Over a 6-day larval duration, optimal larval trajectories actually visited the surface less often when food was limited, since they struggled to gather sufficient energy for metamorphosis over such a short period (Figures S3.7 and S3.8, row D). Finally, over a larval duration of 80 days, optimal trajectories visited the surface for at least the first 10 days of dispersal, and returned frequently to avoid starvation (Figures S3.7 and S3.8, row E).

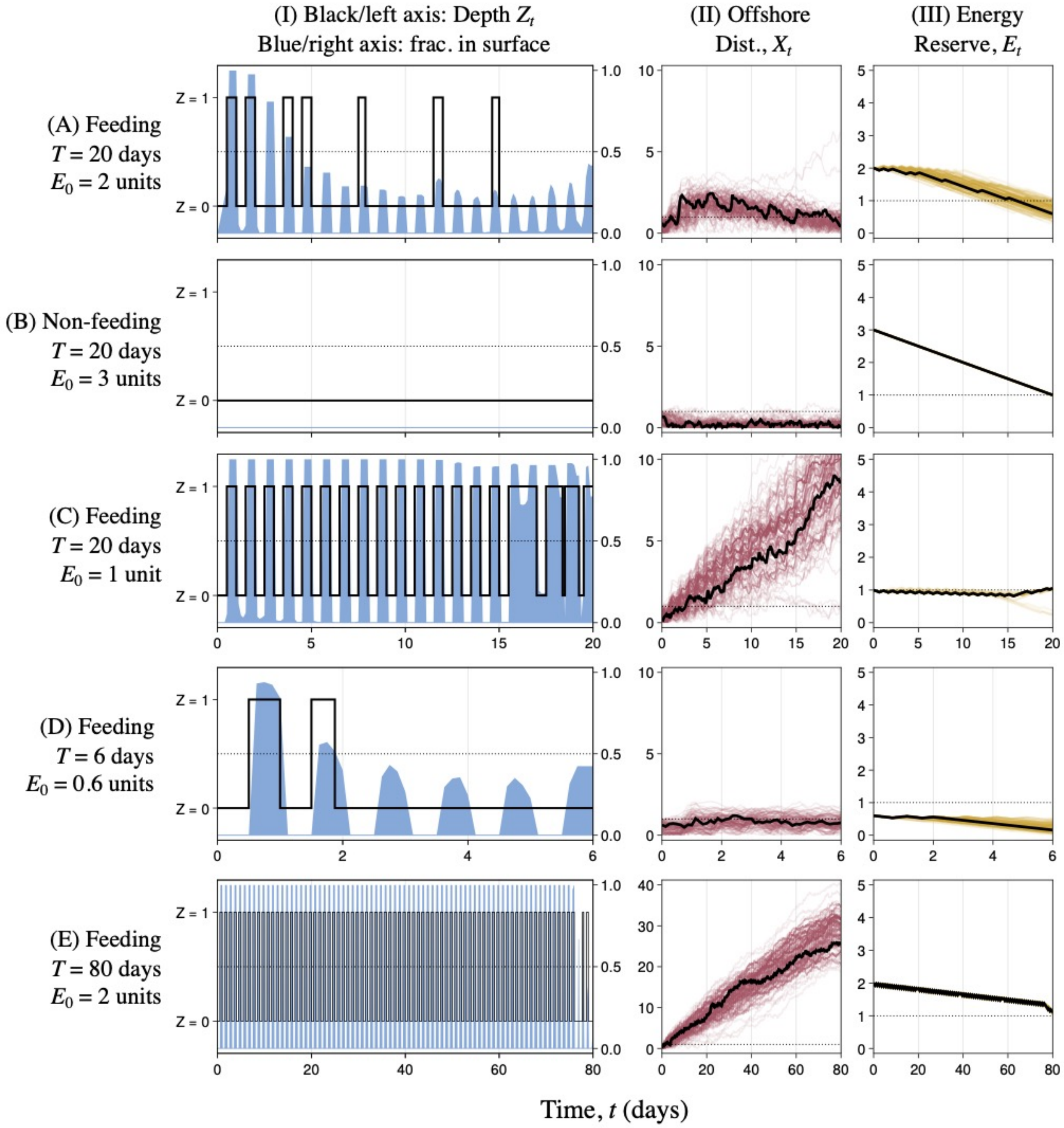

Figure S3.7: *Diurnal predation, upwelling, low food/small surplus.* Analogous to Figure S3.1.

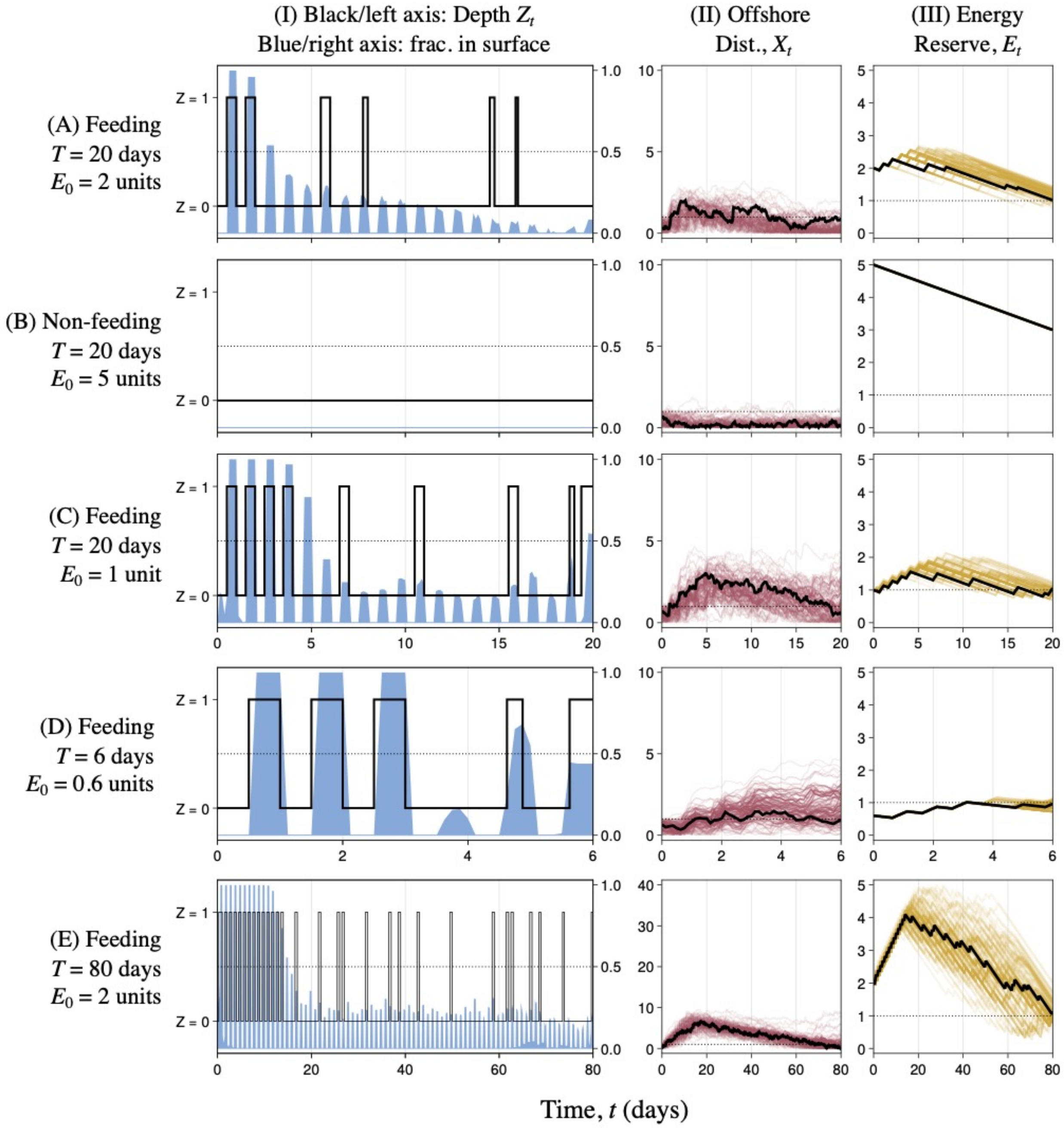

Figure S3.8: *Diurnal predation, upwelling, high food/large surplus.* Analogous to Figure S3.2.

4 Comparing Optimal and Archetypal Swimming Behaviors

4.1 Alternate Versions of Figures 4 and 5

Figures 4 and 5 in the main text compare the Trajectory Scores obtained by optimal swimming behaviors and the DVM, OVM, and Hybrid archetypes against passive drifting for various parameter values. Those figures clearly illustrate that vertical swimming can offer advantages over passive drifting, but perhaps the more relevant comparison is between the optima and the archetypes. We created Figures S4.1 and S4.2 to support this comparison; they are analogous to main text Figures 4 and 5, respectively. In these figures, each panel compares Trajectory Scores from passive drifting or a behavioral archetype against those associated with the optimal behaviors. As noted in the text, the DVM archetype actually performed better than the optimum for larvae with a 20-day larval duration in an environment with weak upwelling, limited food, and diurnal predation (Figure S4.1B.II). Mathematically, this should not have been possible. It is probably not due to sampling error, since DVM remained super-optimal when we re-ran the code for this figure. We suspect this is an artifact due to interpolation- and discretization-related numerical errors in our optimization algorithm.

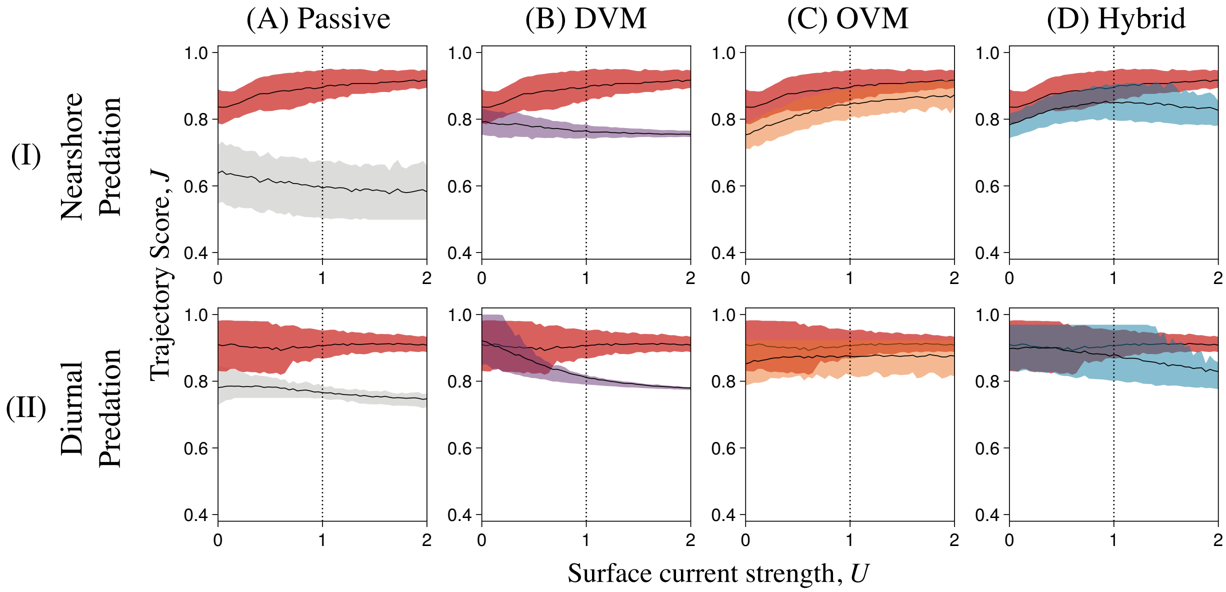

Figure S4.1: Relationships between Trajectory Scores, J, and upwelling current velocities, U, for optimal vertical swimming behaviors (red in all panels), (A) passive drifting (gray), (B) the DVM archetype (purple), (C) the OVM archetype (orange), and (D) the Hybrid archetype (blue). We considered both the nearshore and diurnal predation schemes (rows I and II, respectively). Parameters besides U were held at the default values in main text Table 1. At $U = 0$ ($U = 1$, vertical dotted lines), conditions were identical to those in main text Figures 3A and C (B and 3).

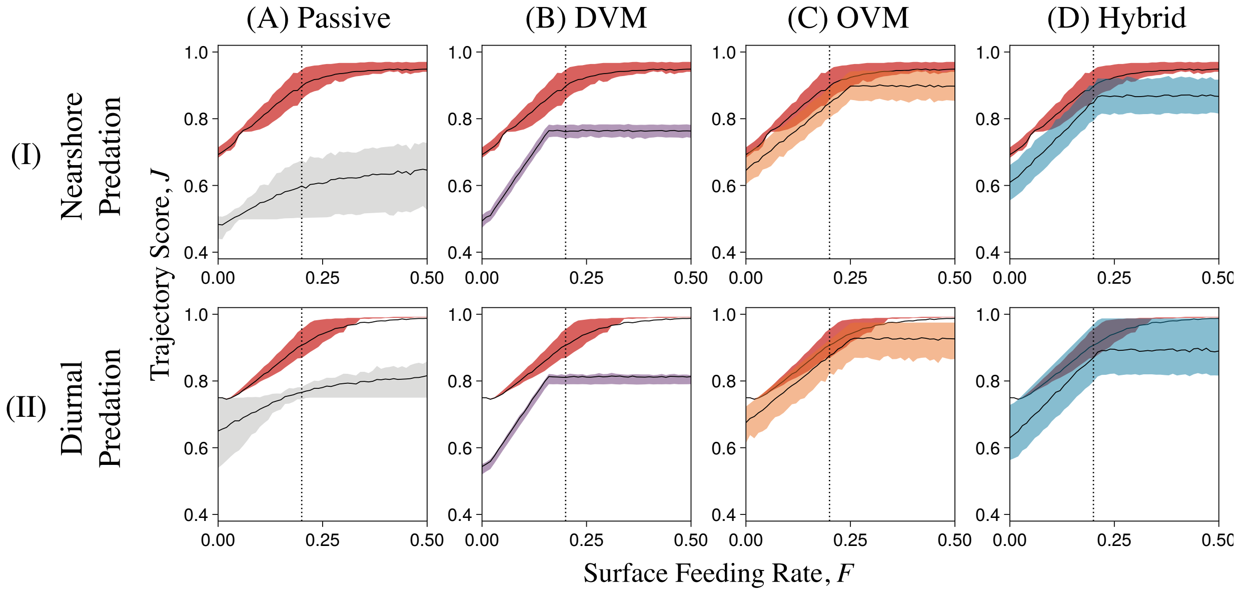

Figure S4.2: Relationships between Trajectory Scores, J, and feeding rate (a proxy for food abundance), F, for optimal vertical swimming behaviors (red in all panels), (A) passive drifting (gray), (B) the DVM archetype (purple), (C) the OVM archetype (orange), and (D) the Hybrid archetype (blue). We considered both the nearshore and diurnal predation schemes (rows I and II, respectively). Parameters besides F were held at the default values in main text Table 1. At $F = 0.2$ (vertical dotted lines), conditions were identical to those in Figure 3B and D.

4.2 Different Biological Scenarios

We repeated the analyses in Sections 3.3 of the main text under two alternate biological scenarios. While the main text considered feeding larvae with a larval duration of 20 days spawned with enough energy for maintenance throughout development, here we considered nonfeeding larvae spawned with enough energy for maintenance and metamorphosis and feeding larvae spawned with insufficient energy for maintenance, both with 20-day larval durations.

4.2.1 Nonfeeding Larvae

Trajectory Scores J depended on U almost identically for feeding and nonfeeding larvae using the DVM, OVM, and Hybrid behavioral archetypes (compare main text Figure 4 to Figure S4.3, columns B-D). Trajectory Scores were slightly greater for nonfeeding larvae because larvae were spawned with enough energy for maintenance and metamorphosis, and almost always finished dispersal with $E_T \approx 1$ or greater. Passive drifting was more successful for nonfeeding larvae on average, also because simulated larvae were not responsible for gathering food (Figure S4.3). For passive drifting, Trajectory Scores were less varied for nonfeeding larvae than feeding

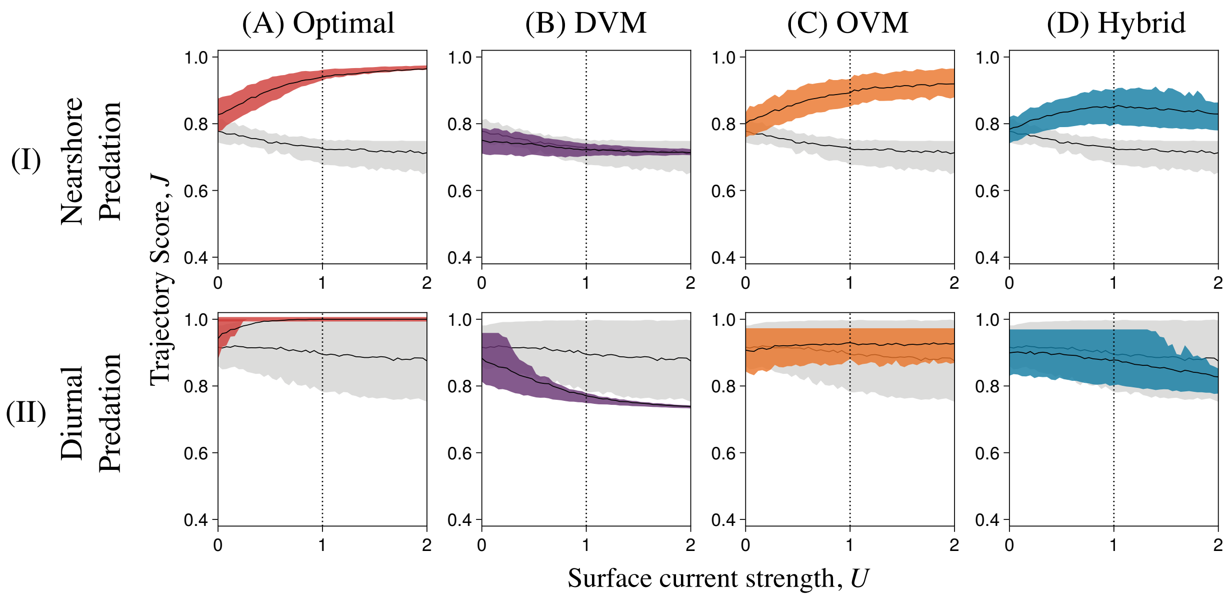

Figure S4.3: Relationships between Trajectory Scores, J, and upwelling current strength, U, for optimal vertical swimming behaviors (red in all panels), (A) passive drifting (gray), (B) the DVM archetype (purple), (C) the OVM archetype (orange), and (D) the Hybrid archetype (blue) for **nonfeeding** larvae. We considered both the nearshore and diurnal predation schemes (rows I and II, respectively). Parameters besides U were held at the default values for nonfeeding larvae in Table 1. At $U = 0$ ($U = 1$, vertical dotted lines), conditions were identical to those in main text Figures 3A and C (B and D).

larvae given nearshore predation, since Scores were not affected by randomness in the amount of food gathered (Figures 4 and S4.3, row I). With diurnal predation, Trajectory Scores were more varied for nonfeeding than feeding larvae, perhaps because for nonfeeding larvae the negative effects of visiting the surface (predation and offshore transport) were not canceled out by the benefits of feeding (Figures 4 and S4.3, row I). Finally, in nearshore predation, Trajectory Scores due from swimming optimally increased with respect to current strength U, since upwelling allowed larvae to advect away from nearshore predators and then toward the nearshore habitat. In diurnal predation, Trajectory Scores were identically 1 for U greater than about 0.2—as noted in Section 3.2.2, nonfeeding larvae in strong advection had no incentive to leave the bottom layer.

Rather than varying food abundance, we varied the size of the energy surplus with which nonfeeding larvae were spawned, S. Recall that $E_0 = GT + S$ where G was the rate of energy use for maintenance, so that larvae were spawned with sufficient energy for maintenance during development. Since metamorphosis cost 1 energy unit and nonfeeding larvae had no means of acquiring additional energy, we only considered values of $S \geq 1$. Due to the small estimated cost of vertical migrations, Trajectory Scores were completely unaffected by S for all behavioral archetypes considered. The only exception was when $S \approx 1$ for DVM, where frequent vertical

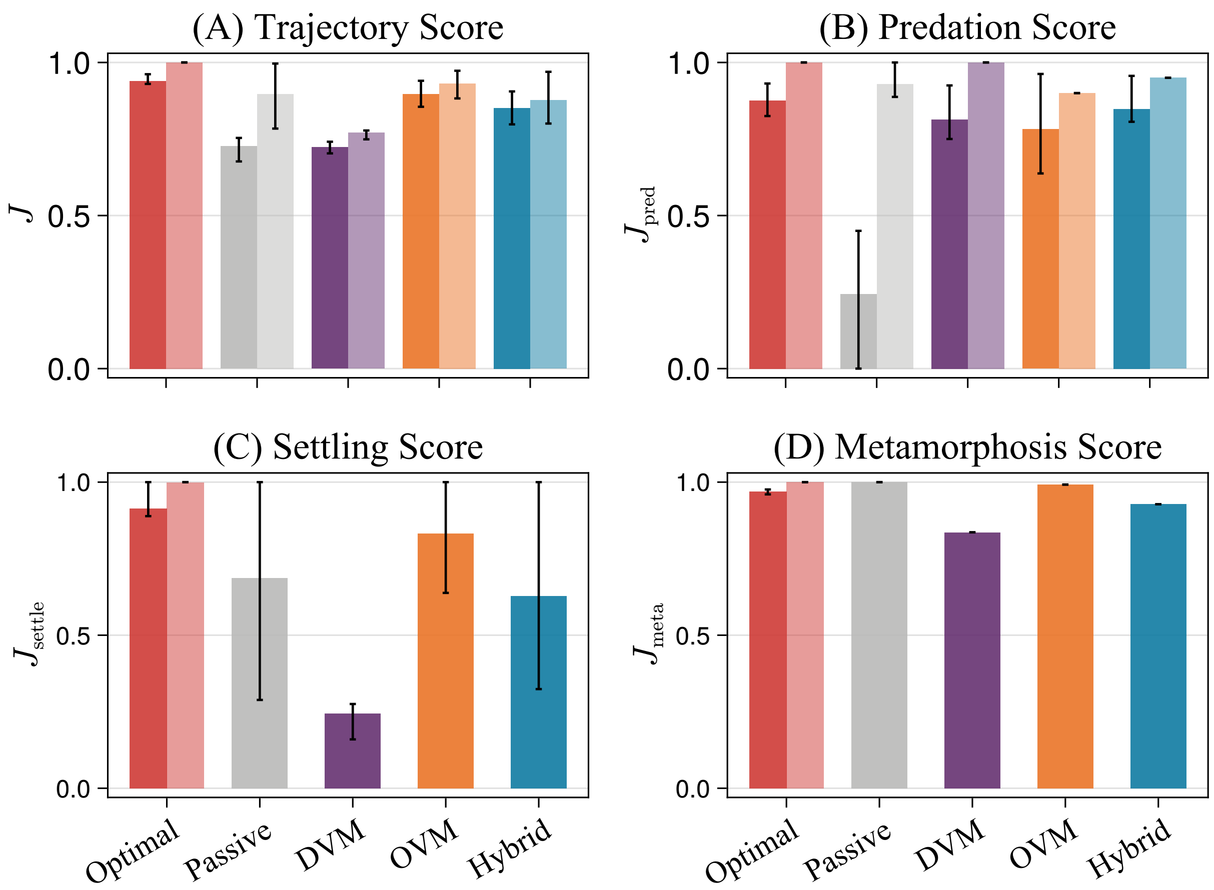

Figure S4.4: Success of the optimal swimming policy, passive drifting, and the DVM, OVM, and Hybrid archetypes with respect to (A) survival through metamorphosis, (B) avoiding predation, (C) settling close to shore, and (D) settling with enough energy for metamorphosis, all for **nonfeeding** larvae with the default parameters in Table 1. Bars represent mean scores, with dark (light) bars corresponding with the nearshore (diurnal) predation schemes. Settling and Metamorphosis Scores did not depend on predation schemes, except that optimal behaviors were different for each scheme. Error bars represent interquartile ranges.

migrations resulted in E_T slightly under 1 and slightly lower Trajectory Scores.

The five behaviors we considered were similarly suitable for feeding and nonfeeding larvae for helping larvae avoid predation, settle close to shore, settle with adequate energy for metamorphosis, and survive from spawning through metamorphosis. Nutritional mode only affected the Metamorphosis and total Trajectory Scores (Figures 6 and S4.4). Of the three archetypes we considered, OVM produced the greatest Trajectory Scores on average, followed by Hybrid and then DVM (Figure S4.4A). OVM was slightly less successful than the other behaviors at predator avoidance (Figure S4.4B), but best allowed larvae to settle close to shore with sufficient energy for metamorphosis (Figures S4.4C-D). Unlike for feeding larvae, passive drifting was similarly suitable compared

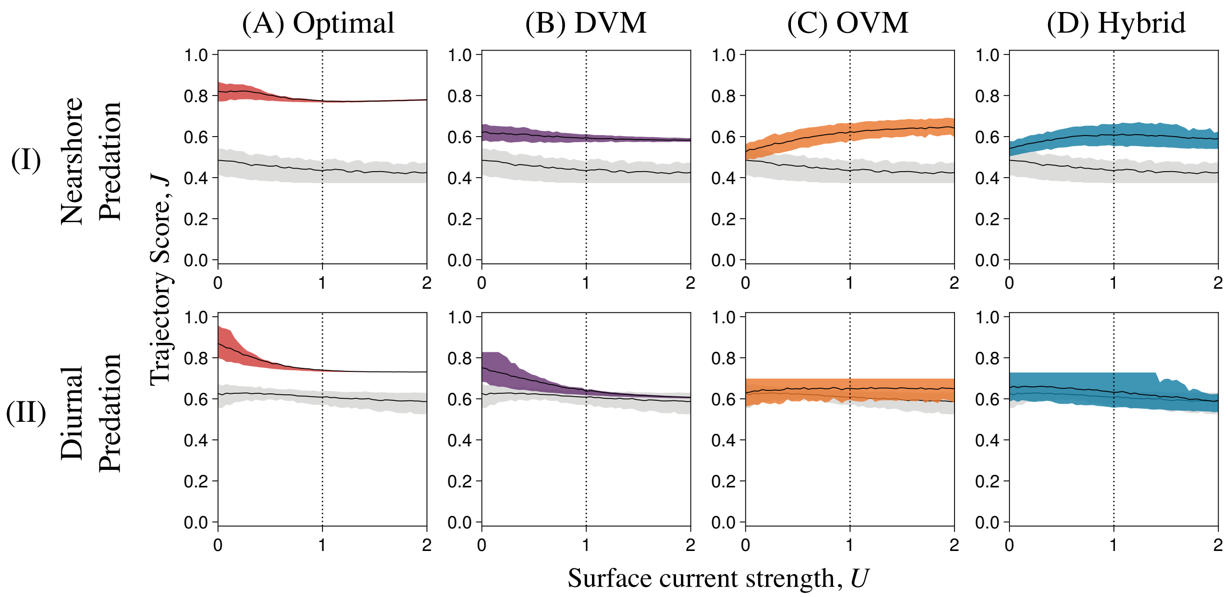

Figure S4.5: Relationships between Trajectory Scores, J, and upwelling current strength, U, for optimal vertical swimming behaviors (red in all panels), (A) passive drifting (gray), (B) the DVM archetype (purple), (C) the OVM archetype (orange), and (D) the Hybrid archetype (blue) for **feeding larvae spawned with insufficient energy for maintenance**, $E_0 = 1$. We considered both the nearshore and diurnal predation schemes (rows I and II, respectively). Parameters besides E_0 and U were held at the default values in Table 1. At $U = 0$ ($U = 1$, vertical dotted lines), conditions were identical to those in main text Figures 3A and C (B and D).

with prescribed archetypes because it required no energy expenditure on swimming: passively drifting larvae spawned with $E_0 = GT + 1$ energy units always completed dispersal with $E_T = 1$ energy units, receiving Metamorphosis Score $J_{\text{meta}} = 1$.

4.2.2 Spawning with Insufficient Energy for Maintenance

The main difference between this case and the one considered in the main text is that here, larvae were able to experience starvation during a 20-day larval duration. This was because initial energy was set to $E_0 = 1$, rather than $E_0 = GT = 2$. This difference in starting energy did not qualitatively affect the relationship between upwelling strength U and Trajectory Scores J for the DVM, OVM, and Hybrid vertical swimming archetypes (Figures 4B-D and S4.5B-D). However, Trajectory Scores for optimal behaviors decreased with U for larvae spawned with insufficient energy (Figure S4.5A) but were generally non-decreasing with U for larvae spawned with sufficient energy (Figure 4A). This was because for larvae spawned with insufficient energy, stronger currents created a greater conflict between food-gathering and avoiding offshore advection. This was particularly evident under low food conditions, as in row C of Figures S3.3 and S3.7.

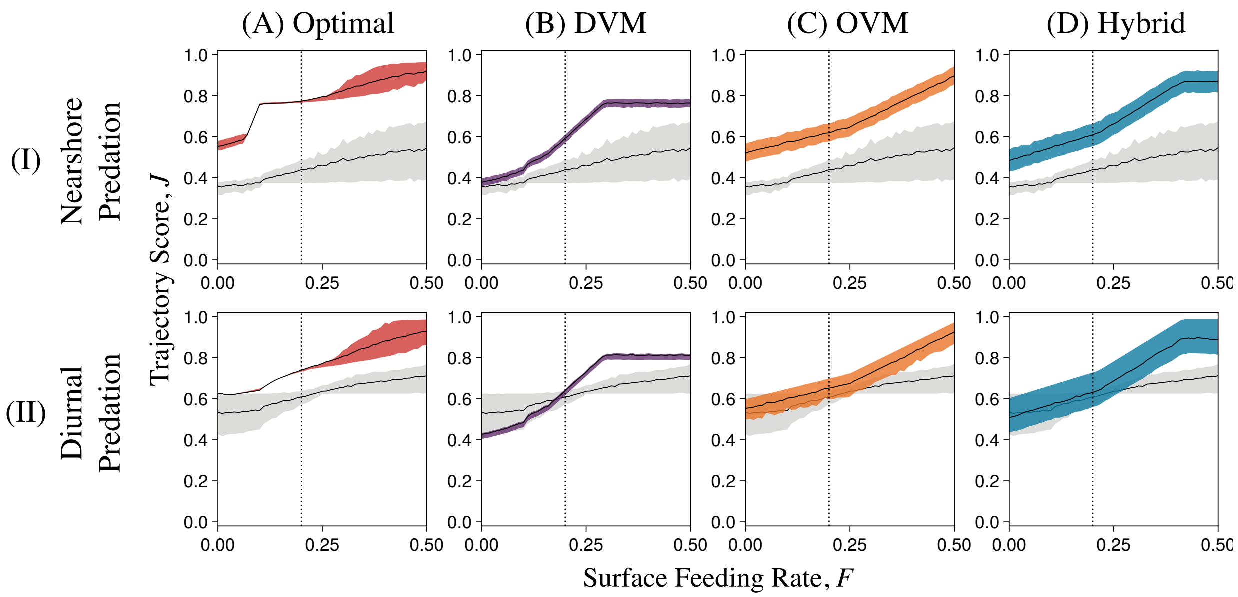

Figure S4.6: Relationships between Trajectory Scores, J, and feeding rate (a proxy for food abundance), F, for optimal vertical swimming behaviors (red in all panels), (A) passive drifting (gray), (B) the DVM archetype (purple), (C) the OVM archetype (orange), and (D) the Hybrid archetype (blue) for **feeding larvae spawned with insufficient energy for maintenance**, $E_0 = 1$. We considered both the nearshore and diurnal predation schemes (rows I and II, respectively). Parameters besides E_0 and F were held at the default values in Table 1. At $F = 0.2$ (vertical dotted lines), conditions were identical to those in Figure 3B and D.

Trajectory Scores were more sensitive to food abundance, F, in this scenario than when larvae were spawned with sufficient energy for maintenance (Figures 5 and S4.6). With sufficient energy, relationships between F and J plateaued for each behavioral archetype at the first value of F where larvae could easily settle with $E_T \geq 1$. With insufficient energy, a second such transition occurred at the first value of F at which larvae could avoid starvation for the entirety of development (for example, see Figure S4.6D). This increased sensitivity suggested that producing larvae with insufficient energy for maintenance would be a risky strategy in environments where food abundance fluctuates over time.

Feeding larvae spawned with insufficient energy for maintenance were unlikely to return to shore with sufficient energy for metamorphosis under the upwelling, low food conditions we considered (Figure S4.7C-D). Optimal behaviors prioritized starvation avoidance and settling with sufficient energy together over settling close to shore alone (Figures S4.7C-E), resulting in trajectories that often ended far offshore (see examples in row C of Figures S3.3 and S3.7). The DVM, OVM, and Hybrid archetypes received similar mean Trajectory Scores (Figure S4.7A), since any behavior that successfully returned larvae to shore (e.g., OVM and Hybrid) failed to gather energy for metamorphosis, and vice versa.

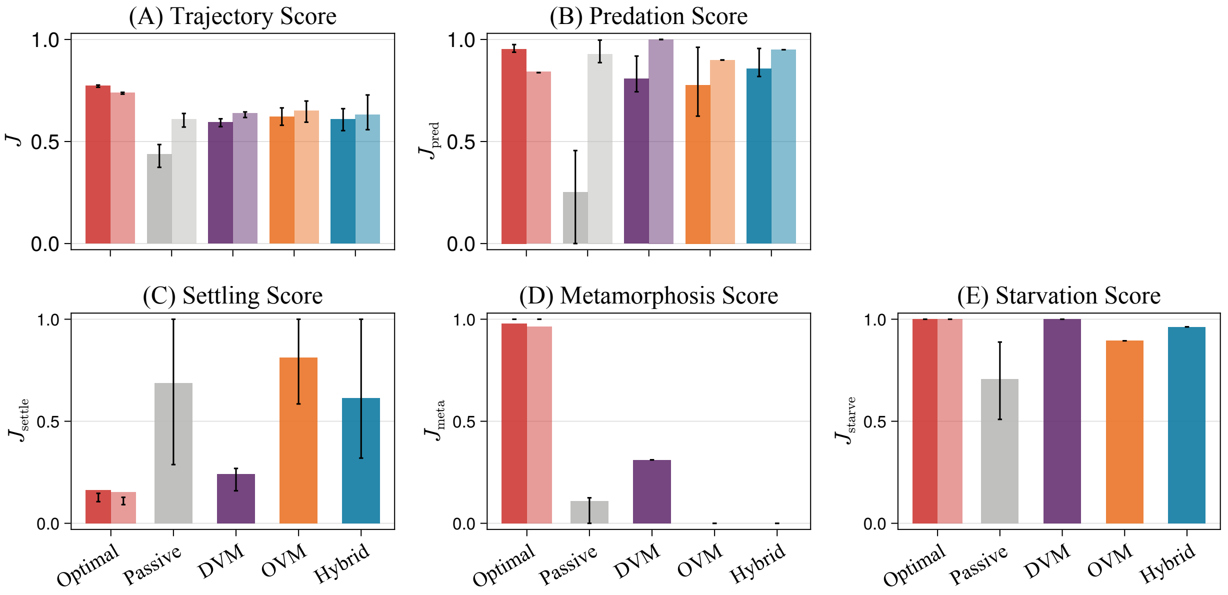

Figure S4.7: Success of the optimal swimming policy, passive drifting, and the DVM, OVM, and Hybrid archetypes with respect to (A) survival through metamorphosis, (B) avoiding predation, (C) settling close to shore, and (D) settling with enough energy for metamorphosis, all for **feeding larvae spawned with insufficient energy for maintenance**. Bars represent mean scores, with dark (light) bars corresponding with the nearshore (diurnal) predation schemes. Settling and Metamorphosis Scores did not depend on predation schemes, except that optimal behaviors were different for each scheme. Error bars represent interquartile ranges; note that some cases, mean Scores fell outside of these ranges.

4.3 Variations on Passive Drifting and DVM

Small details of how larval swimming behaviors are modeled can have unexpectedly large impacts on predictions of larval transport (Meyer et al, 2021; Sundelöf and Jonsson, 2012). Therefore, we repeated our analysis of behavioral archetypes for two alternative formulations of diel vertical migrations (DVM) and one alternative formulation of passive drifting. The DVM archetype in the main text featured larvae visiting the surface each night for six hours each night. We shall refer to this behavior as the DVM-6 archetype in this Supplement. The two alternatives visited the surface for three and 12 hours each night, and will be referred to as the DVM-3 and DVM-12 archetypes, respectively. The passive drifting behavior in the main text was described above Supplement 2 above. As an alternative, we considered the approximation from Meyer et al (2021), in which larvae switched between layers after exponentially distributed residence times. On average, visits to the surface and bottom layers lasted one and 13 hours, respectively. We will refer to these as the Main and Alternate forms of Passive Drifting.

The Alternate version of Passive Drifting resulted in similar Trajectory Scores, J, to the Main version with

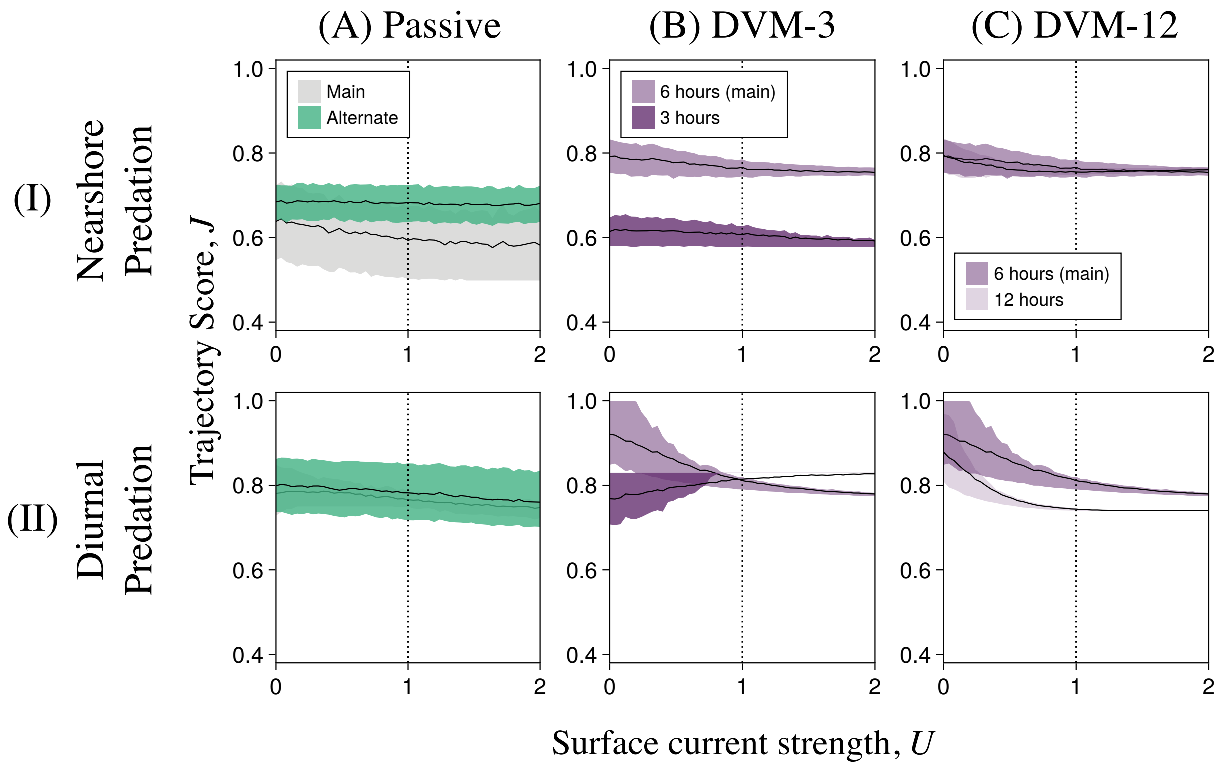

Figure S4.8: Relationships between Trajectory Scores, J, and upwelling current strength, U, for (A) the Main and Alternate versions of Passive Drifting (gray and green, respectively), (B) DVM-3 and DVM-6 (dark and medium purple, respectively), and (C) DVM-6 and DVM-12 (medium and light purple, respectively) for feeding larvae with a 20-day larval duration. We considered both the nearshore and diurnal predation schemes (rows I and II, respectively). Parameters besides U were held at the default values in Table 1. At $U = 0$ ($U = 1$, vertical dotted lines), conditions were identical to those in main text Figures 3A and C (B and D).

diurnal predation, but higher Scores with nearshore predation (Figure S4.8A and S4.10A). This was due to the Alternate version's more frequent surface visits that resulted in greater transport away from the dangerous nearshore habitat (Figure S4.10B). In both predation schemes, Trajectory Scores for both versions of Passive Drifting decreased slightly with current strength U, but were largely insensitive (Figure S4.8A). In contrast, Trajectory Scores increased with respect to food abundance F, and the Alternate version was more sensitive to F (Figure S4.9). Finally, we noted substantially more variance in Metamorphosis Scores for the Main version of Passive Drifting compared with the Alternate version (Figure S4.10D), reflecting greater variability for the Main version in how much time trajectories spend feeding in the surface layer.

The DVM-3 archetype responded quite differently to changes in current strength U and food abundance F from the DVM-6 (main text) and DVM-12 archetypes, which were qualitatively similar (Figures S4.8B-C and S4.9B-C). This difference can be understood using the analytical framework of Meyer et al (2021). For a generic

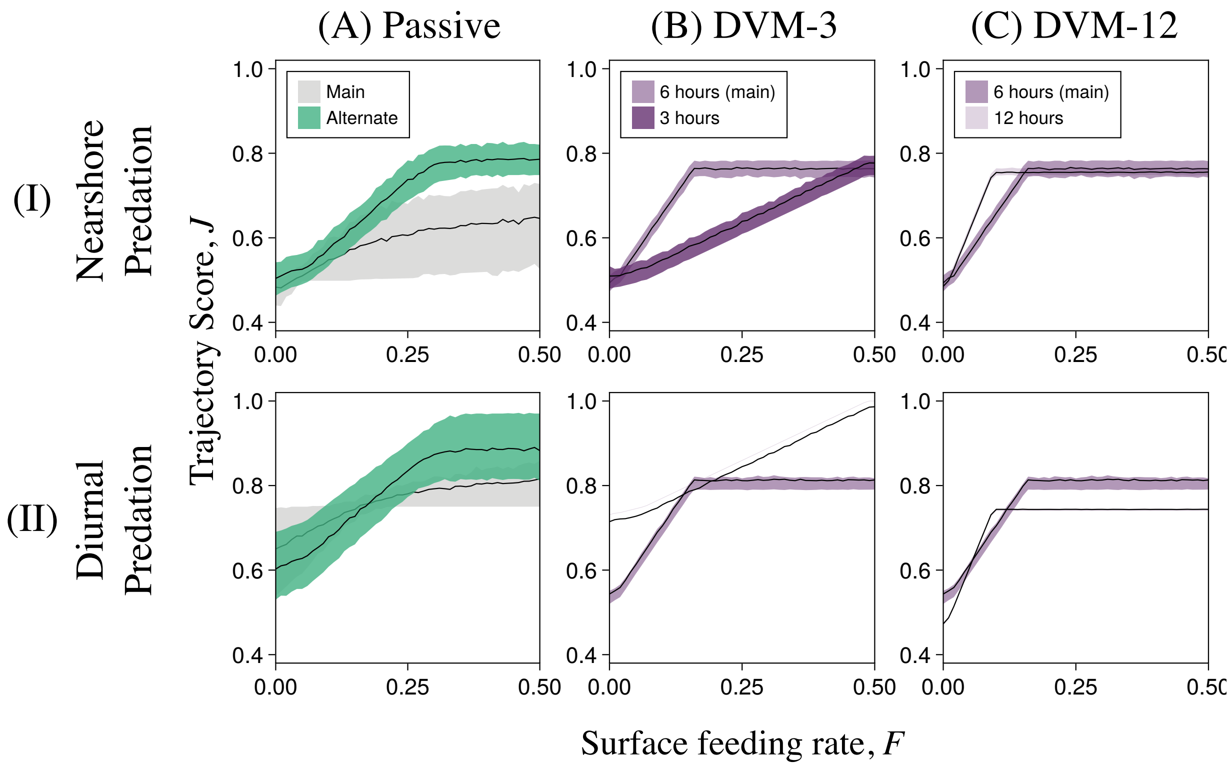

Figure S4.9: Relationships between Trajectory Scores, J, and feeding rate (a proxy for food abundance), F, for (A) the Main and Alternate versions of Passive Drifting (gray and green, respectively), (B) DVM-3 and DVM-6 (dark and medium purple, respectively), and (C) DVM-6 and DVM-12 (medium and light purple, respectively) for feeding larvae with a 20-day larval duration. We considered both the nearshore and diurnal predation schemes (rows I and II, respectively). Parameters besides F were held at the default values in Table 1. At $F = 0.2$ (vertical dotted lines), conditions were identical to those in Figure 3B and D.

DVM behavior where larvae spend fraction $n \in [0, 24]$ hours in the surface each night, we expect larvae to travel about

$$\text{Expected daily displacement (EDD)} = \frac{nU}{24} - \alpha\left(1 - \frac{n}{24}\right)U$$

habitat widths offshore per day. For the default $\alpha = 1/4$, this simplifies to

$$\text{EDD} = \frac{(5n - 24)U}{96}, \tag{4.1}$$

which is negative (implying onshore transport) when $n < 4.8$ hours. Thus, DVM-3 resulted in mean-onshore transport and significant nearshore retention, while DVM-6 and DVM-12 resulted in mean-offshore transport and

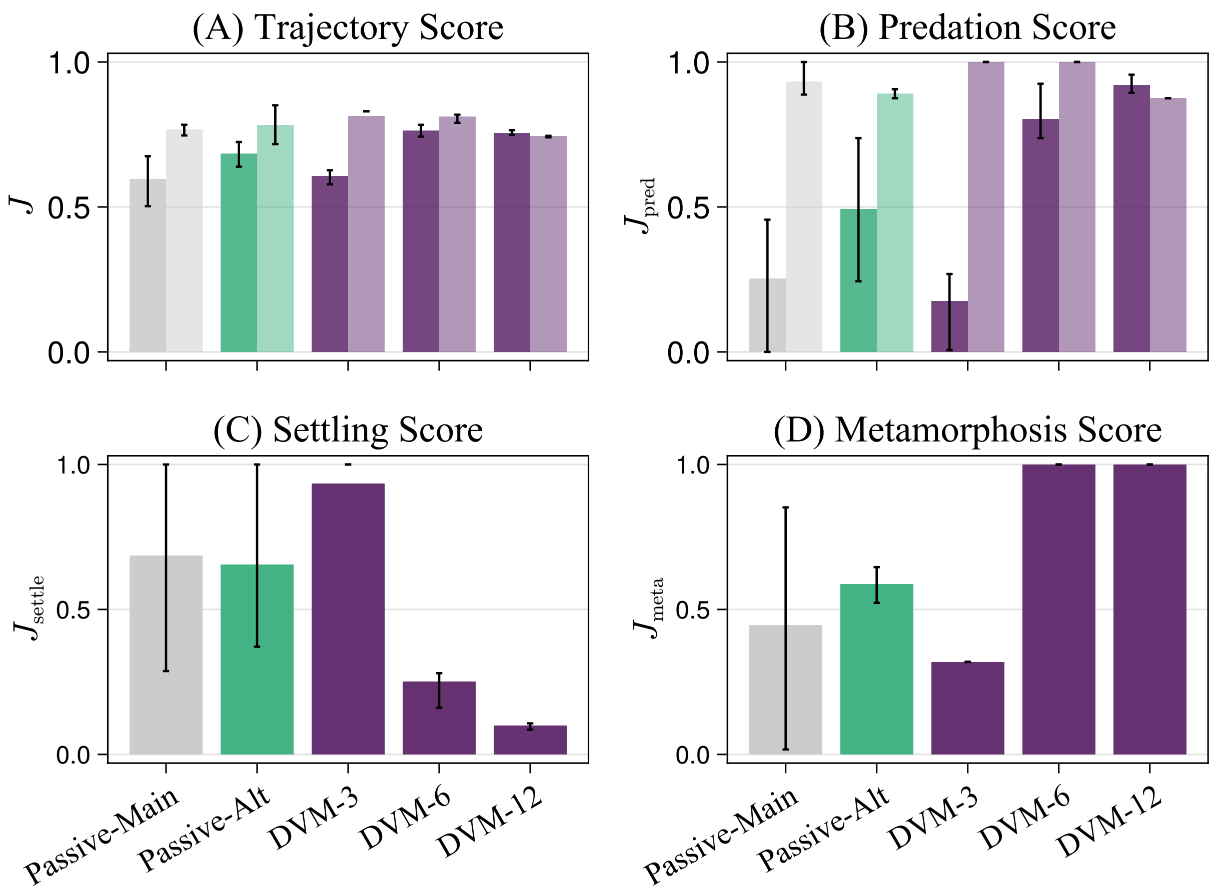

Figure S4.10: Success of Main and Alternate versions of passive drifting and the DVM-3, DVM-6, and DVM-12 swimming archetypes with respect to (A) survival through metamorphosis, (B) avoiding predation, (C) settling close to shore, and (D) settling with enough energy for metamorphosis. All parameters were fixed at the default values in Table 1. Bars represent mean scores, with dark (light) bars corresponding with the nearshore (diurnal) predation schemes. Settling and Metamorphosis Scores did not depend on predation schemes, except that optimal behaviors were different for each scheme. Error bars represent interquartile ranges.

greater sensitivity to conditions in the surface and offshore.

With nearshore predation, the DVM-3 archetype received lower Trajectory Scores on average than the DVM-6 and DVM-12 archetypes due to nearshore retention (Figures S4.8 and S4.9 row I, and Figure S4.10A-B). With diurnal predation, nearshore retention was advantageous because it promoted settling close to shore; this was reinforced by a stronger upwelling current. Consequently, Trajectory Scores for DVM-3 increased with U under diurnal predation, while Trajectory Scores for DVM-6 and DVM-12 decreased with U under the same conditions (Figure S4.8B-C). On the other hand, Trajectory Scores for DVM-3 were more sensitive to food abundance F over the range $0 \leq F \leq 0.5$ than they were for DVM-6 and DVM-12 (Figure S4.9B-C), since

larvae performing DVM-3 had shorter windows in which to feed during development.

Overall, Trajectory Scores for the DVM archetypes were fairly similar, particularly given diurnal predation (Figure S4.10A). This was because larvae performing these behaviors experienced a trade-off between settling close to shore (which requires avoiding the surface's offshore current) and gathering energy for metamorphosis (which requires feeding in the surface). This is shown in Figures S4.10C-D. Biologically, this suggests that different variants of DVM could be beneficial in different contexts.

5 Weights of the Trajectory Score

In the main text, we weighed predator avoidance, starvation avoidance, settling close to shore, and settling with energy for metamorphosis equally, $p_i = 1/4$. This was a conservative assumption, since the relative contributions of predation, starvation, offshore wastage, and settling with inadequate energy to total larval mortality are generally considered poorly resolved and highly variable in nature (Morgan, 1995; Rumrill, 1990). In this supplement, we show that weighting one requirement more heavily than the others resulted in mostly subtle changes to the optimal swimming behaviors we computed. We remade Figure 3 from the main text using three alternate sets of weights that prioritized either predator avoidance (Figure S5.1), settling close to shore (Figure S5.2), or settling with more energy (Figure S5.3). We set the weight $p_{\text{starve}} = 0$ for these cases because, as in Figure 3 in the main text, our default parameters were chosen such that starvation was nearly impossible. For conciseness, Table S5.1 summarizes key differences between the optimal larval trajectories in Figure 3 and those with different weights in Figures S5.1-S5.3.

Table S5.1: Key differences between optimized larval trajectories when one of predator avoidance, settling close to shore, or settling with more energy is prioritized compared with when all three are equally important, as in the main text. Parameters besides the weights p_i were fixed at the default values in main text Table 1.

	**Predator Avoidance**<br>$p_{\text{pred}} = 0.8$<br>$p_{\text{settle}} = p_{\text{meta}} = 0.1$	**Settling Site**<br>$p_{\text{settle}} = 0.8$<br>$p_{\text{pred}} = p_{\text{meta}} = 0.1$	**Settling Energy**<br>$p_{\text{meta}} = 0.8$<br>$p_{\text{pred}} = p_{\text{settle}} = 0.1$
Nearshore predation, Still water	None (Figs. 3A and S5.1A).	Shorter, more frequent surface visits (Figs. 3A and S5.2A).	None (Figs. 3A and S5.3A).
Nearshore predation, Upwelling	Longer initial surface visit, at cost of settling nearshore (Figs. 3B and S5.1B).	Many short surface visits to maintain nearshore position (Figs. 3B and S5.2B).	Surface visits at end of dispersal to maintain $E_T \geq 1$, at cost of settling nearshore (Figs. 3B and S5.3B).
Diurnal predation, Still water	None (Figs. 3C and S5.1C).	If needed, remain in surface during daylight near end of dispersal to try to achieve onshore diffusion (Figs. 3C and S5.2C).	None (Figs. 3C and S5.3C).
Diurnal predation, Upwelling	None (Figs. 3D and S5.1D).	None (Figs. 3D and S5.2D).	More frequent nocturnal surface visits for feeding, at cost of nearshore settling (Figs. 3D and S5.3D).

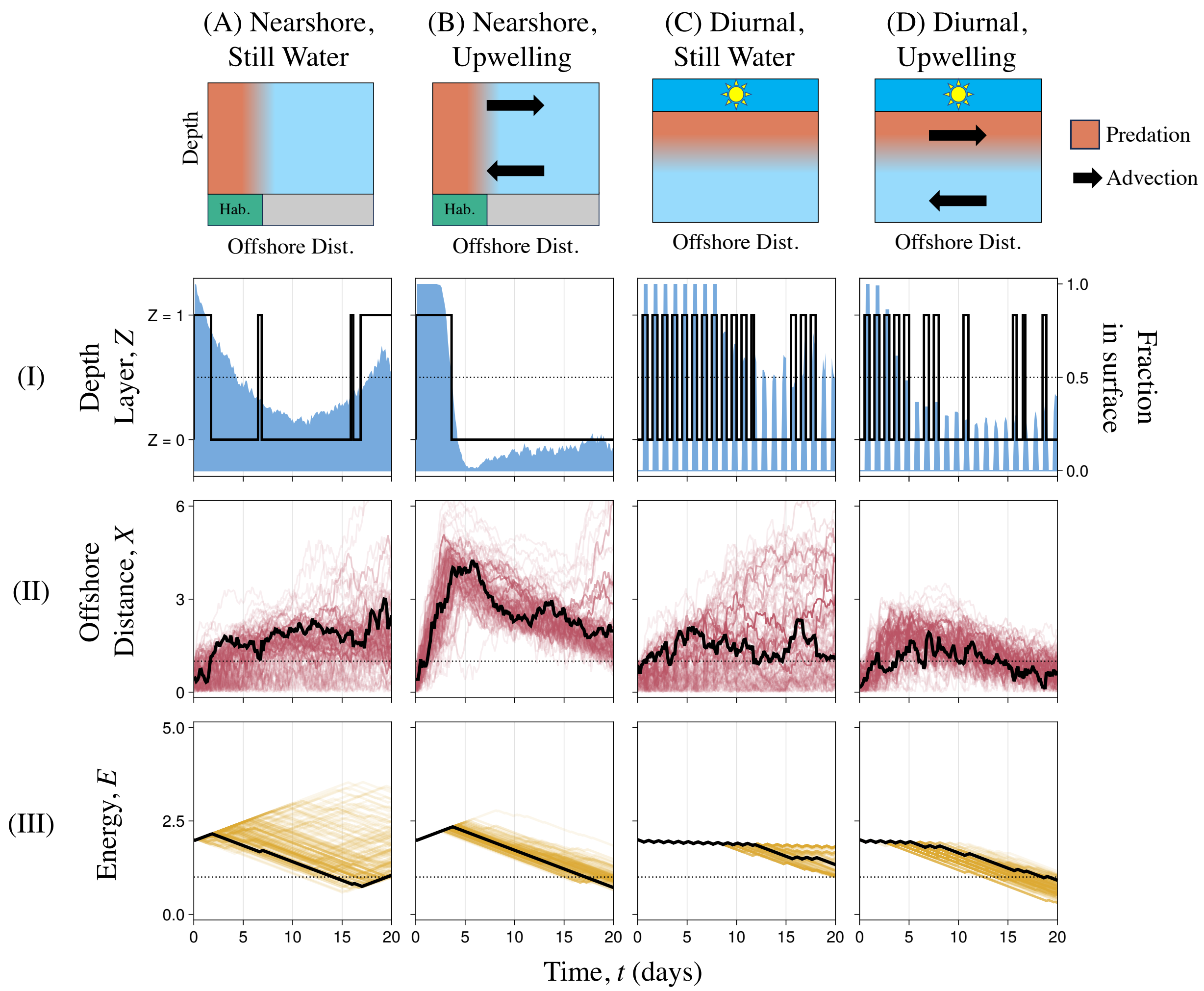

Figure S5.1: Optimized trajectories of simulated larvae subject to (A) nearshore predation in still water, (B) nearshore predation with upwelling, (C) diurnal predation in still water, and (D) diurnal predation with upwelling, with **predator avoidance prioritized over settling site and energy**. Diagrams in the top row illustrate these environmental conditions. Within each column, the solid black curves show the (I) depth, Z_t, (II) offshore distance, X_t, and (III) energy reserve, E_t, of a median simulated larva under each set of conditions. The blue shading in (I) shows the fraction of several optimized larvae in the surface over time (right axis). The red and yellow curves in (II) and (III) each show 100 additional optimized trajectories X_t and E_t, respectively.

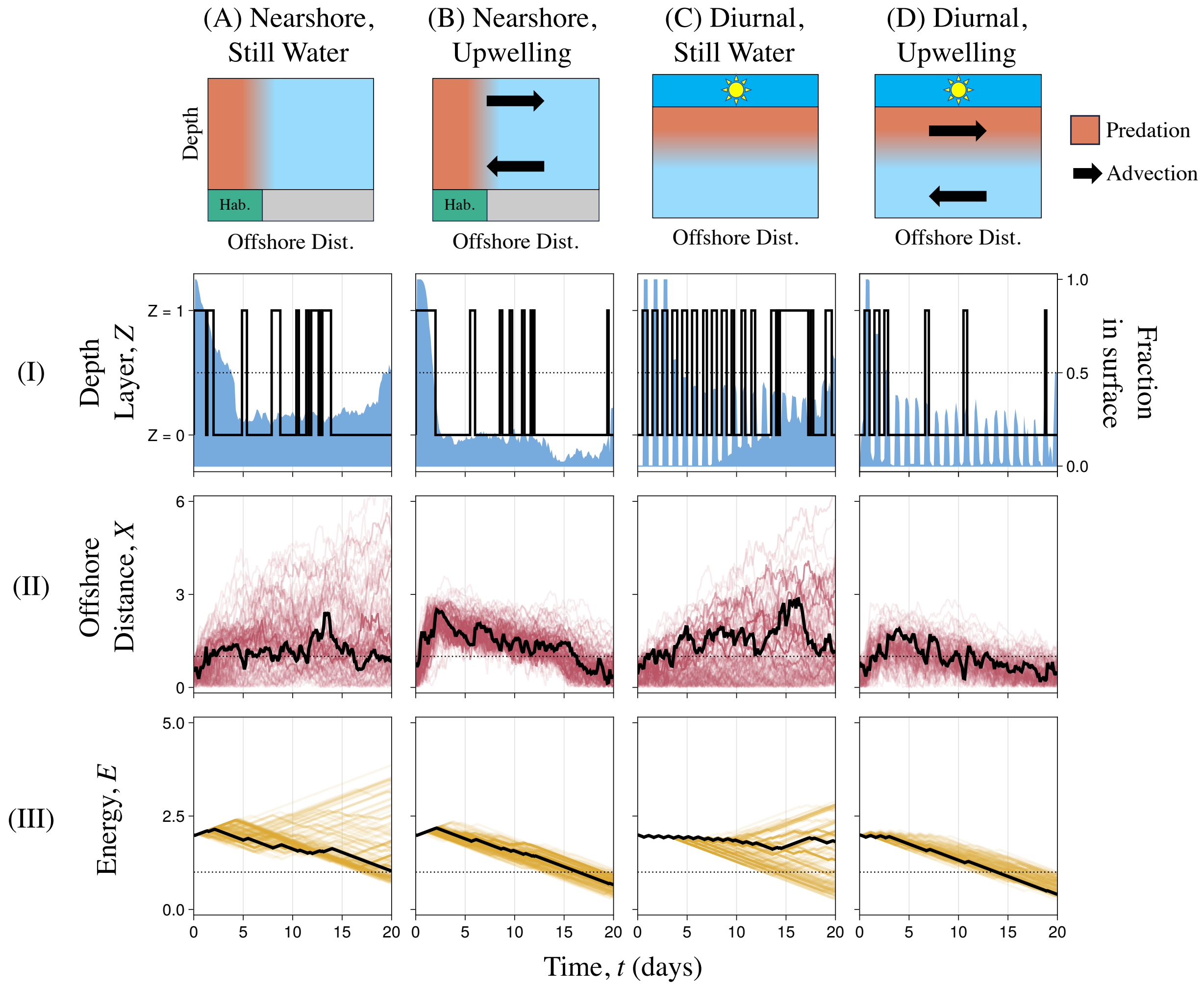

Figure S5.2: Optimized trajectories of simulated larvae subject to (A) nearshore predation in still water, (B) nearshore predation with upwelling, (C) diurnal predation in still water, and (D) diurnal predation with upwelling, with **settling close to shore prioritized over predator avoidance and settling energy**. Diagrams in the top row illustrate these environmental conditions. Within each column, the solid black curves show the (I) depth, Z_t, (II) offshore distance, X_t, and (III) energy reserve, E_t, of a median simulated larva under each set of conditions. The blue shading in (I) shows the fraction of several optimized larvae in the surface over time (right axis). The red and yellow curves in (II) and (III) each show 100 additional optimized trajectories X_t and E_t, respectively.

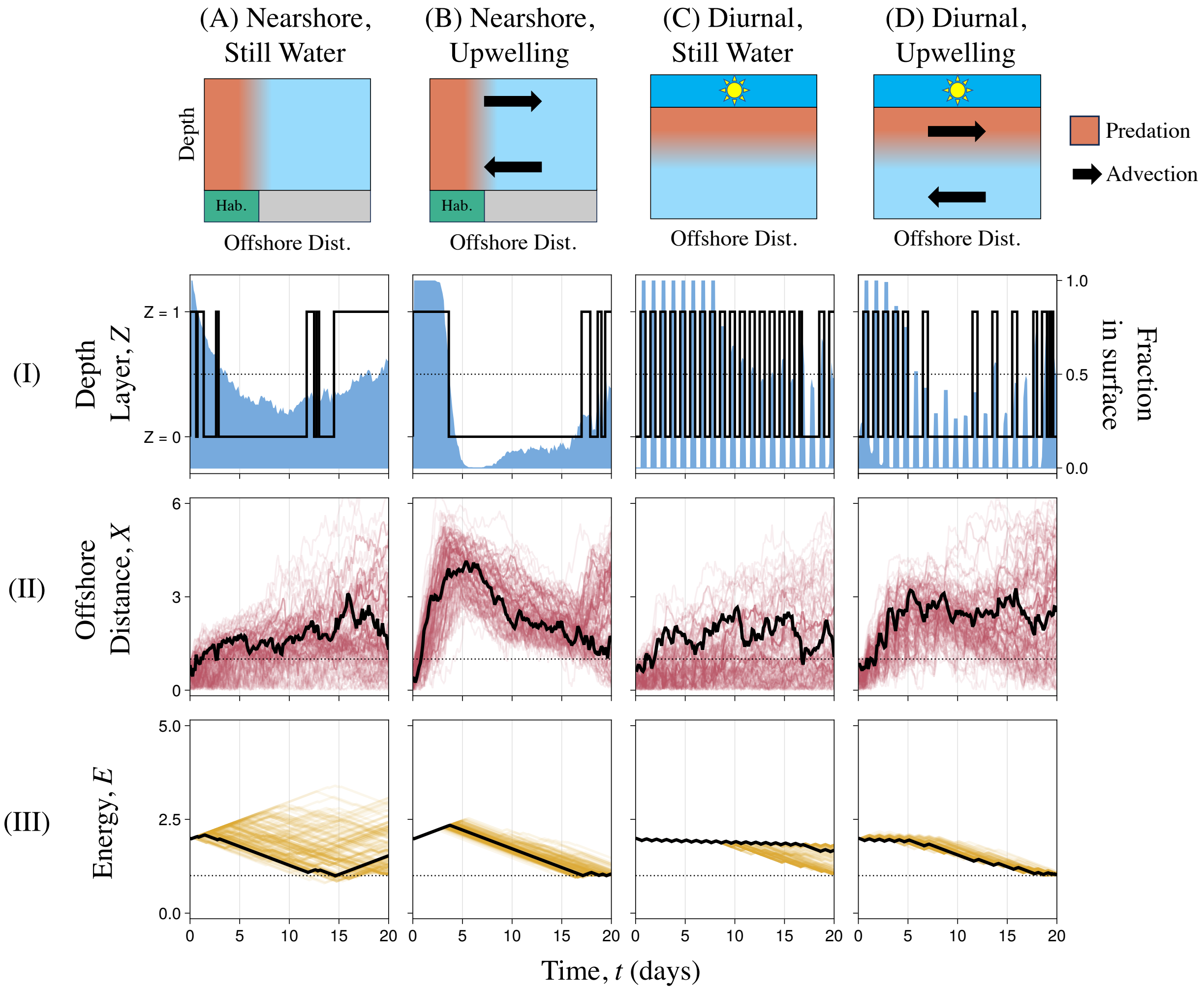

Figure S5.3: Optimized trajectories of simulated larvae subject to (A) nearshore predation in still water, (B) nearshore predation with upwelling, (C) diurnal predation in still water, and (D) diurnal predation with upwelling, with **energy at settling prioritized over predator avoidance and settling close to shore**. Diagrams in the top row illustrate these environmental conditions. Within each column, the solid black curves show the (I) depth, Z_t, (II) offshore distance, X_t, and (III) energy reserve, E_t, of a median simulated larva under each set of conditions. The blue shading in (I) shows the fraction of several optimized larvae in the surface over time (right axis). The red and yellow curves in (II) and (III) each show 100 additional optimized trajectories X_t and E_t, respectively.

6 Additional Simulations of Optimal Larvae

In main text Figure 3 (and other figures like it), we were able to visualize offshore distance, X_t, and energy content, E_t, for many optimized larval trajectories under various conditions (e.g., red curves in row II and yellow curves in row III). However, because $Z_t \in \{0, 1\}$, we were only able to visualize one depth trajectory per scenario (black curve in row I), along with the average of many such trajectories (blue in row 1). For completeness, Figure S6.1 shows additional simulations of Z_t in the four scenarios considered in main text Figure 3: a species with feeding larvae spawned with $E_0 = GT$ and a 20-day larval duration in environments with limited food, either nearshore or diurnal predation, and either still water or upwelling circulation.

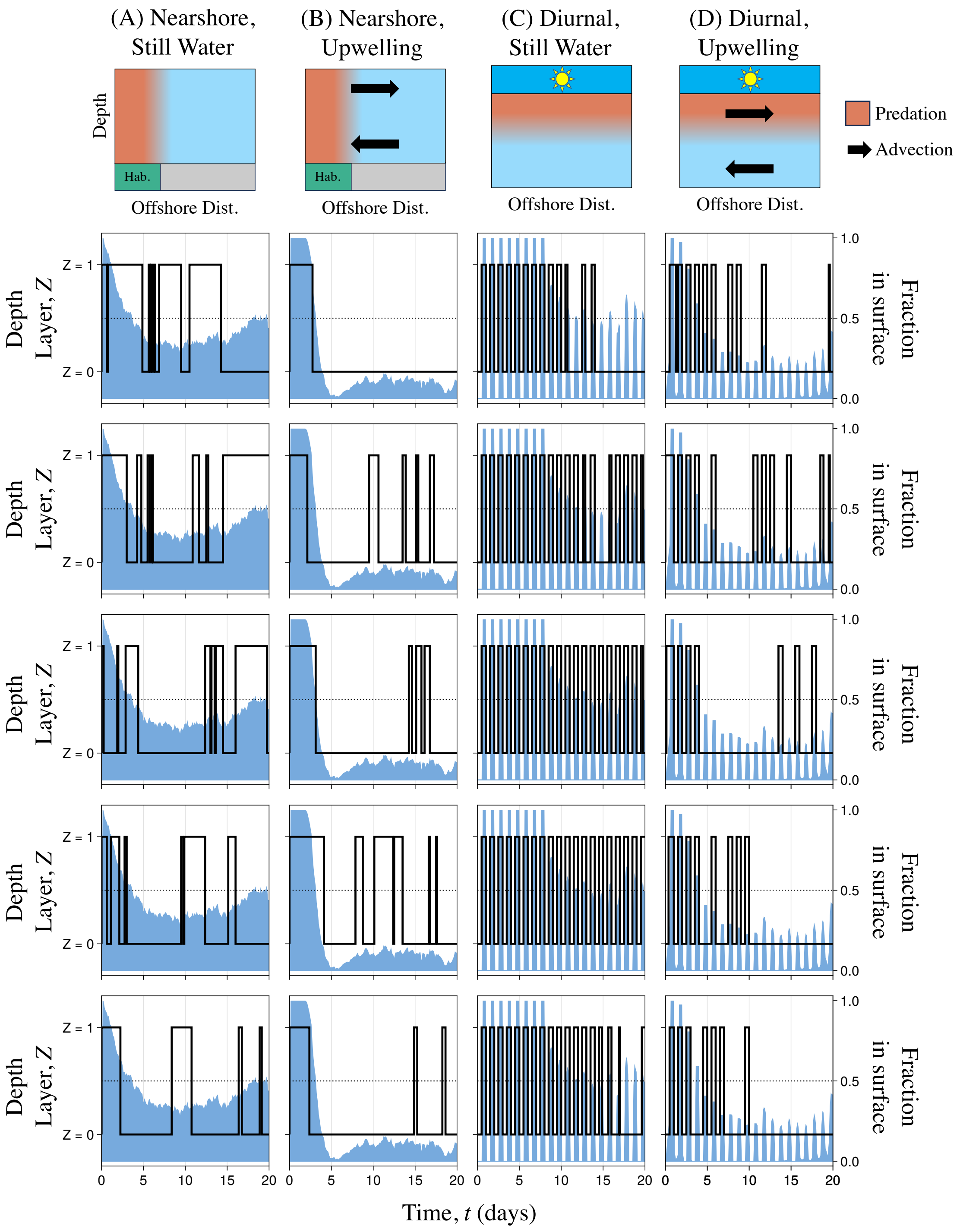

Figure S6.1: Five optimal depth trajectories, Z_t, for simulated larvae with the default parameters in main text Table 1 and (A) nearshore predation in still water, (B) nearshore predation with upwelling, (C) diurnal predation in still water, and (D) diurnal predation with upwelling. Example trajectories are shown in black (left axis), while the average of all depth trajectories in each case (e.g., the fraction of optimal larvae in the surface, right axis) is shown in blue.

References

Barton ED, Inall ME, Sherwin TJ, Torres R (2001) Vertical structure, turbulent mixing and fluxes during lagrangian observations of an upwelling filament system off northwest iberia. Progress in Oceanography 51:249–267

Chia FS, Buckland-Nicks J, Young CM (1984) Locomotion of marine invertebrate larvae: a review. Canadian Journal of Zoology 62:1205–1222

Hamilton P, Jr MR (1978) A numerical model of the depth-dependent, wind-driven upwelling circulation on a continental shelf. Journal of Physical Oceanography 8:437–457

Haskell WZ, Kadko D, Hammond DE, Knapp A, Prokopenko MG, Berelson WM, Capone DG (2015) Upwelling velocity and eddy diffusivity from 7be measurements used to compare vertical nutrient flux to export poc flux in the eastern tropical south pacific. Marine Chemistry 168:140–150

Largier JL (2003) Considerations in estimating larval dispersal distances from oceanographic data. Ecological Applications 13(1):S71–S89

Lucas MI, Walker G, Holland DL, Crisp DJ (1979) An energy budget for the free-swimming and metamorphosing larvae of *Balanus balanoides* (Crustacea: Cirripedia). Marine Biology 55:221–229

Meyer AD, Hastings A, Largier JL (2021) Larvae of coastal marine invertebrates enhance their settling success or benefits of planktonic development – but not both – through vertical swimming. Oikos 130:2260–2278

Morgan SG (1995) Ecology of Marine Invertebrate Larvae, CRC Press, chap 9. Life and Death in the Plankton: Larval Mortality and Adaptation, pp 279–321. Marine Science Series

Nickols KJ, Gaylord B, Largier JL (2012) The coastal boundary layer: Predictable current structure decreases alongshore transport and alters scales of dispersal. Marine Ecology Progress Series 464:17–35

Nickols KJ, White JW, Largier JL, Gaylord B (2015) Marine population connectivity: Reconciling large-scale dispersal and high self-retention. The American Naturalist 185(2):196–211

Rasmuson LK (2013) The Biology, Ecology and Fishery of the Dungeness crab, *Cancer magister*, Advances in Marine Biology, vol 65, Elsevier Ltd., chap 3, pp 95–148

Rumrill SS (1990) Natural mortality of marine invertebrate larvae. Ophelia 32(1-2):163–198

Shanks AL (1995) Ecology of Marine Invertebrate Larvae, CRC Press, chap 10. Mechanisms of Cross-Shelf Dispersal of Larval Invertebrates and Fish, pp 323–367. Marine Science Series

Shanks AL (2009) Pelagic larval duration and dispersal distance revisited. Biological Bulletin 216:373–385

Shanks AL, Grantham BA, Carr MH (2003) Propagule dispersal distance and the size and spacing of marine reserves. Ecological Applications 13(1):S159–S169

Sprung M (1984a) Physiological energetics of mussel larvae (*Mytilus edulis*). ii. Food uptake. Marine Ecology Progress Series 17:295–305

Sprung M (1984b) Physiological energetics of mussel larvae (*Mytilus edulis*). iii. Respiration. Marine Ecology Progress Series 18:171–178

Sundelöf A, Jonsson PR (2012) Larval dispersal and vertical migration behaviour – a simulation study for short dispersal times. Marine Ecology 33:183–193

Thiyagarajan V, Harder T, Qiu JW, Qian PY (2003) Energy content at metamorphosis and growth rate of the early juvenile barnacle *Balanus amphitrite*. Marine Biology 143:543–554

Waldron HN, Probyn TA (1991) Short-term variability during an anchor station study in the southern benguela upwelling system: Nitrogen supply to the euphotic zone during a quiscent phase in the upwelling cycle. Progress in Oceanography 28(1-2):153–166

Wendt DE (2000) Energetics of larval swimming and metamorphosis in four species of bugula (Bryozoa). Biological Bulletin 198:346–356
